# A peroxidase-derived ligand that induces *Fusarium graminearum* Ste2 receptor-dependent chemotropism

**DOI:** 10.3389/fcimb.2023.1287418

**Published:** 2024-01-04

**Authors:** Pooja S. Sridhar, Vinicio Vasquez, Fanny Monteil-Rivera, John S. Allingham, Michele C. Loewen

**Affiliations:** ^1^ Department of Biomedical and Molecular Sciences, Queen’s University, Kingston, ON, Canada; ^2^ National Research Council of Canada, Aquatic and Crop Resources Development, Montreal, QC, Canada; ^3^ National Research Council of Canada, Aquatic and Crop Resources Development, Ottawa, ON, Canada

**Keywords:** chemotropism, peroxidase, ligand, GPCR, plant-pathogen interaction, carbohydrate

## Abstract

**Introduction:**

The fungal G protein-coupled receptors Ste2 and Ste3 are vital in mediating directional hyphal growth of the agricultural pathogen *Fusarium graminearum* towards wheat plants. This chemotropism is induced by a catalytic product of peroxidases secreted by the wheat. Currently, the identity of this product, and the substrate it is generated from, are not known.

**Methods and results:**

We provide evidence that a peroxidase substrate is derived from *F. graminearum* conidia and report a simple method to extract and purify the *Fg*Ste2-activating ligand for analyses by mass spectrometry. The mass spectra arising from t he ligand extract are characteristic of a 400 Da carbohydrate moiety. Consistent with this type of molecule, glycosidase treatment of *F. graminearum* conidia prior to peroxidase treatment significantly reduced the amount of ligand extracted. Interestingly, availability of the peroxidase substrate appears to depend on the presence of both *Fg*Ste2 and *Fg*Ste3, as knockout of one or the other reduces the chemotropism-inducing effect of the extracts.

**Conclusions:**

While further characterization is necessary, identification of the *F. graminearum*-derived peroxidase substrate and the *Fg*Ste2-activating ligand will unearth deeper insights into the intricate mechanisms that underlie fungal pathogenesis in cereal crops, unveiling novel avenues for inhibitory interventions.

## 1 Introduction

G protein-coupled receptors (GPCRs) are a family of seven transmembrane domain (7-TM) proteins that mediate cellular responses towards a wide range of extracellular stimuli, including but not limited to hormones, sugars, and peptides. When a GPCR is activated by its corresponding ligand, it undergoes a conformational change enabling its interaction with intracellular proteins that initiate signaling cascades leading to a cellular response (Latorraca et al., 2017; Hilger et al., 2018; Weis and Kobilka, 2018). It is now known that GPCRs can bind an assortment of ligands and exhibit a multitude of functionally unique conformations (Hilger et al., 2018). This enables a single receptor to activate different signaling pathways that give different biological outcomes; a phenomenon termed ‘biased signaling’. While GPCR research has mainly focused on mammalian receptors, the past few years have seen a growing interest in the fungal pheromone receptors Ste2 and Ste3, which have been implicated in virulence of fungi that infect agricultural plants.

The fungal GPCRs Ste2 and Ste3 were first identified as pheromone-sensing receptors in the yeast *Saccharomyces cerevisiae* (*Sc*Ste2p and *Sc*Ste3p) (Hagen et al., 1986; Blumer et al., 1988). *Sc*Ste2p and *Sc*Ste3p are expressed on the surfaces of opposite mating type cells in *S. cerevisiae* and are activated by the α- and a-pheromones, respectively (Schrick et al., 1997; Jones and Bennett, 2011; Alvaro and Thorner, 2016). Binding of these pheromones to *Sc*Ste2p and *Sc*Ste3p triggers the well characterized pheromone-response MAPK signaling pathway and leads to mating of the two cells (Schrick et al., 1997; Alvaro and Thorner, 2016). *Sc*Ste2p has also been reported to exhibit alternate functionalities, dictated by factors such as pheromone gradients (Segall, 1993; Brizzio et al., 1996; Elia, 1996; Barkai et al., 1998) and localization of the *Sc*Ste2p receptor to the mating projection (Jackson et al., 1991). Mutations in the ligand-binding residues of the receptor alter G protein-mediated MAPK signaling and lead to different impacts on diploid zygote formation (Dube and Konopka, 1998; Shi et al., 2007; Choudhary and Loewen, 2016a).

The role of Ste2 has been more enigmatic in higher fungi, particularly in those that do not require mating for reproduction. However, several studies have shed light on novel functions for Ste2 and its counterpart Ste3 in pathogenic fungi. Recently, Ste2 has been shown to mediate directed hyphal growth, or chemotropism, of the fungal pathogens *Fusarium oxysporum* (Turrà et al., 2015), *Fusarium graminearum* (Sridhar et al., 2020) and *Verticillium dahliae* (Vangalis et al., 2022) towards their host plants. The a-pheromone receptor Ste3 in *F. graminearum* has similarly been implicated in chemotropism and pathogenicity on wheat (Sharma et al., 2022). Similar to what is observed in Ste2 in *F. graminearum*, deletion of Ste3 results in a complete elimination of chemotropism towards the wheat plant. In the non-pathogenic fungus *Trichoderma reesei*, the Ste2 and Ste3 orthologs, Hpr1 and Hpr2, respectively, mediate chemotropic sensing of plant root exudates (Hinterdobler et al., 2021). Ste2- and Ste3-mediated chemotropism in these fungi is induced by the activity of peroxidases secreted by their host plants. Furthermore, Ste2 and Ste3 have important roles in the control of conidial germination in a cell density-dependent manner in *F. oxysporum* (Vitale et al., 2019).

Previous work in *F. graminearum* has emphasized that the wheat peroxidases only induce chemotropism when they are catalytically active, implying that they convert a fungal- or wheat-derived substrate into a chemoattractant that is recognized by the Ste2 receptor (*Fg*Ste2) (Sridhar et al., 2020). The identity of this *Fg*Ste2-activating ligand remains to be determined. The class III peroxidase family in plants, to which the wheat peroxidases belong, is typically involved in building and reinforcing the plant cell wall through polymerization of phenolic monomers of lignin (Hiraga, 2001; Passardi et al., 2004; Almagro et al., 2009; Cosio and Dunand, 2009). They can concurrently reduce hydrogen peroxide while oxidizing a range of phenolic and non-phenolic substrates (Pandey et al., 2017). Peroxidases also promote the degradation of polysaccharides through the generation of hydroxyl radicals in the presence of NADH (Schweikert et al., 2002).

Here, we have now demonstrated that an *Fg*Ste2-activating ligand is produced from a peroxidase substrate that originates from *F. graminearum* conidia and we have established a simple method to extract and enrich this ligand. Peroxidase treatment of *F. graminearum* strains lacking either *Fg*Ste2 or *Fg*Ste3 produces extracts that are unable to induce a chemotropic response, illustrating a link between expression of the two receptors and the synthesis or availability of the peroxidase substrate. Liquid chromatography coupled with mass spectrometry (LC-MS) analysis of the peroxidase-treated *F. graminearum* extracts identified a 400 Da species as a ligand candidate. Its elution time from the LC column and oxygenated nature indicate that this molecule is most likely a carbohydrate. In line with this characterization, treatment of *F. graminearum* conidia with glycosidases resulted in a decrease in the amount of ligand that could be extracted. Moreover, RNA sequencing analysis of *Fg*Ste2-depleted *F. graminearum* showed downregulation of several cell wall and carbohydrate-related genes. Together, these findings allude to a complex mechanism of chemotropic growth wherein the fungal GPCRs and the host peroxidase are both involved in producing the chemoattractant.

## 2 Materials and methods

### 2.1 Fungal strains, culture conditions and maintenance

A list of *F. graminearum* and *S. cerevisiae* strains used in this study are presented in **Table 1** and **Table 2**, respectively. The oligonucleotides used in strain construction and confirmation are listed in **Table 3**. Strains of *F. graminearum* were maintained, cultured, and stored as previously described (Sridhar et al., 2020).

**Table 1:** List of *F. graminearum* strains used in this study.

Strain	Genotype	Gene function	Reference
GZ-3639	wild type		Dr. Susan McCormick (USDA)
*Fgste2*Δ	*STE2::HPH*	GPCR	Sridhar et al. (Sridhar et al., 2020)
*Fgste2*Δ+*STE2*	*STE2::HPH;STE2:GEN*	GPCR	Sridhar et al. (Sridhar et al., 2020)
*Fgste3Δ*	*STE3::HPH*	GPCR	Sharma et al. (Sharma et al., 2022)
*Fgmgv1Δ*	*MGV1::HPH*	MAPK	Rampitsch et al. (Rampitsch et al., 2011)

**Table 2:** List of *S. cerevisiae* strains used in this study.

Strain	Genotype	Reference
BY4741 YFL026W	*MATa his3Δ1 leu2Δ0 met15Δ0 ura3Δ0 ΔSTE2*	Winzeler et al.
BY4741 YFL026W+*Fg*Ste2	*MATa his3Δ1 leu2Δ0 met15Δ0 ura3Δ0 ΔSTE2 FgSTE2-URA3*	This study

**Table 3:** Oligonucleotides used in this study.

Target	F/R	Primer	Sequence (5’→3’)	Product size (bp)
*ScSTE2* gene	F	P1	GATGCGGCTCCTTCATTGAGC	1241
	R	P2	GCTGCCGTATCGGGAGTGTAC	
*FgSTE2* gene	F	P3	CTGCCATTGACCAGGTGC	657
	R	P4	AGATGCCGTTGGTCATGATGAG	

All strains of *S. cerevisiae* used in this study were derived from BY4741 YFL026W, a wild-type strain derived from S288C and lacking the endogenous *ScSTE2* gene (Winzeler et al., 1999; Shi et al., 2009). BY4741 YFL026W was routinely maintained on YPD plates (1% yeast extract, 2% peptone, 2% dextrose, 2% agar) supplemented with 200 µg/mL geneticin.

### 2.2 Plasmid construction

The pYESDEST52 plasmid harbouring the *FgSTE2* gene (*FGSG_02655*) was generated by Gateway cloning (Invitrogen). *FgSTE2* was amplified by polymerase chain reaction using Phusion DNA polymerase. Following this, a second PCR was performed to attach the *attb* ends required for Gateway cloning. The cloning was then performed according to the manufacturer’s instructions. Briefly, 750 ng of amplified *FgSTE2* was mixed with 2 µL BP clonase enzyme and 1.5 µL pDONR221 vector and made up to 20 µL with Tris-EDTA (TE; pH 8.0) buffer. The reaction was incubated overnight at 18°C. The following day, 2 µL of proteinase K was added to the reaction and incubated at 37°C for 30 min. The reaction was then transformed into *E. coli* JM109 cells and positive colonies that grew on kanamycin-containing LB plates were confirmed to have the correct insert by PCR.

For destination cloning into pYES-DEST52, a 1:1 molar ratio of pDONR221::*Fg*Ste2 and empty pYES-DEST52 was mixed with 2 µL of LR clonase enzyme, 4 µL of 5x LR clonase buffer and made up to 20 µL with TE buffer. The reaction was incubated at 25°C for 18 h, followed by a hold at 4°C. The reaction was transformed into *E. coli* JM109 cells, following which positive ampicillin-resistant transformants were confirmed by PCR and restriction digestion. Sanger sequencing was used to validate the correct sequence of the inserted gene.

### 2.3 Generation of *Fg*Ste2-expressing BY4741 YFL026W

The BY4741 YFL026W strain was confirmed to lack the endogenous *ScSTE2* gene by polymerase chain reaction using primers P1 and P2. Transformation of BY4741 YFL026W by electroporation was performed as previously described with minor modifications (Becker and Guarente, 1991). Strain YFL026W was inoculated in 7.5 mL YPD overnight at 30°C with shaking at 180 rpm. Five mL of the overnight culture was inoculated in 50 mL of YPD and grown until an O.D._600_ of 0.5 – 0.7. The cells were harvested by centrifugation at 3400 g for 5 min at 4°C, resuspended in 12.5 mL of permeabilization buffer (100 mM LiAc, 10 mM DTT, 10 mM Tris-HCl, pH 7.5, 1 mM EDTA) and incubated at room temperature for 1 h. Cells were pelleted by centrifugation and washed twice in ice-cold sterile water. The cell pellet was then resuspended in 5 mL of ice-cold 1 M sorbitol. The cells were pelleted again and resuspended in 100 µL of ice-cold 1 M sorbitol. Approximately 1 µg of pYES-DEST52-*FgSTE2* was mixed with the cells and incubated on ice for 5 min. The mixture was transferred to a 0.2 cm electroporation cuvette and pulsed with a BioRad gene pulser using the “eukaryotic” setting. Immediately, 500 µL of ice cold 1 M sorbitol was added and mixed with the contents of the cuvette. Aliquots of 100 µL were plated on Synthetic Dropout (SD) medium containing 2% glucose, 0.66% yeast nitrogen base, 200 mg/mL arginine, 200 mg/mL histidine, 200 mg/mL leucine, 0.2% yeast dropout mix lacking uracil, arginine, leucine and histidine, and 200 µg/mL geneticin. Positive transformants that grew on plates lacking uridine were confirmed by PCR amplification of the *FgSTE2* gene using internal gene primers (P3 and P4).

Expression of *Fg*Ste2 was tested in various galactose-containing media and for different expression times. For overnight expression, cells were inoculated in either YPD, YP-Galactose (YP-Gal; 1% yeast extract, 2% peptone, 2% galactose), or Synthetic Dropout Complete (SDC) medium (2% sucrose, 0.66% yeast nitrogen base, 200 mg/mL arginine, 200 mg/mL histidine, 200 mg/mL leucine, 0.2% yeast dropout mix lacking uracil, arginine, leucine and histidine) supplemented with 2% galactose and incubated at 30°C overnight with shaking at 200 rpm. The following day, the cells were harvested by centrifugation at 3400 g for 10 min and flash frozen until further use.

For checking shorter expression times, cells were inoculated in YPD overnight at 30°C with shaking at 200 rpm. The following day, cells were diluted to an O.D._600_ of 0.1 in YP-Gal or SDC media supplemented with 2% galactose incubated at 30°C with shaking at 200 rpm for 4 hours. Cells were then collected by centrifugation and flash frozen until further use.

### 2.4 Western blotting of *Fg*Ste2 in *S. cerevisiae*

Expression of *Fg*Ste2 was confirmed by Western Blotting using an anti-6xhis antibody as previous described (Sridhar et al., 2020). Briefly, cell pellets were frozen at a ratio of 100 µL of breaking buffer (50 mM sodium phosphate, pH 8.0, 1 mM EDTA, 5% glycerol) supplemented with Complete EDTA-free Protease Inhibitor Cocktail per mL of culture. To the tube, 100 µL of glass beads were added and then cells were lysed for eight cycles of 30 s vortexing followed by 30 sec on ice. To this mixture, Triton X-100 was added to a final concentration of 1% and incubated on a rocker at 4°C for 1 h. The samples were centrifuged at 12,000 *g* for 20 min and the supernatant was transferred to a fresh microfuge tube. A Bradford assay (Bradford, 1976) was performed to quantify total protein concentration.

For the Western blot, 10 µg of total protein was resolved on a 10% SDS-PAGE and subsequently transferred to a polyvinylidene difluoride (PVDF) membrane by wet electroblotting at 400 mA for 2 h. The membranes were blocked for 1 h in 5% (w/v) non-fat dried skim milk in TBST (50 mM Tris, pH 7.5, 150 mM NaCl, 0.05% (v/v) Tween 20) at 4°C. The membranes were subsequently incubated with anti-6x his primary antibody (1:1,000 dilution, ab1187, Abcam). Pierce Enhanced Chemiluminescent substrate was added to the membranes and the emitted light was captured on an Azure C300 imager.

### 2.5 Dilution spot assay

The spot assay for *F. graminearum* conidia was performed as previously described with minor modifications (Yun et al., 2014). Briefly, freshly harvested *F. graminearum* conidia were quantified using a hemocytometer and diluted to 10^7^ conidia/mL in sterile water. Serial dilutions of 10^6^, 10^5^, 10^4^ conidia/mL were then prepared for each strain. One microliter of each suspension was spotted onto PDA plates containing either 0.2 mM H_2_O_2_, 300 µg/mL Calcofluor White (CFW), 1 M sorbitol, or no stressor and incubated at 25°C for 48 hours.

### 2.6 Quantitative chemotropism plate assay

Assessment of chemotropism was performed using the quantitative plate assay as described previously (Sridhar et al., 2020). Briefly, fresh *F. graminearum* macroconidia were mixed with 0.5% (*w/v*) water agar to a final concentration of 2.5 x 10^5^ spores per mL and plated in a Petri dish. A scoring line was drawn down the middle of the plate and two wells were made 5 mm away and parallel to the scoring line. Equal volumes (50 µL) of sterile water and test compound were pipetted into the control well and test well, respectively. Plates were incubated for approximately 14 h at 22°C in the dark. The number of germinating hyphae growing towards the test (N_test_), or control compound (N_cont_) were counted under the Nikon SMZ1000 microscope, and a chemotropic index was calculated as $\text{C}.\text{I}=\frac{Ntest-Ncontrol}{Ntest+Ncontrol}\times 100\%$ .

### 2.7 Preparation of horse radish peroxidase (HRP) solutions

Horse radish peroxidase (Sigma P-8375) was resuspended in sterile deionized water at a concentration ranging from 22-120 µM (1-5.5 mg/mL). This enzyme concentration was verified by measuring the optical density of the resuspension at 403 nm (using an extinction coefficient, ϵ_403_ of 100 M^-1^ cm^-1^).

### 2.8 Peroxidase activity assay

The peroxidase activity assay was performed in 96-well plates at 22°C as described previously (Maehly and Chance, 1954). The formation of the pyrogallol oxidation product (extinction coefficient, ϵ420, 4,400 M^-1^ cm^-1^) was measured spectrophotometrically at 420 nm. To assess the production of hydrogen peroxide by *F. graminearum* conidia, enzyme reactions containing 4 μM HRP, 40 mM pyrogallol, and either 14.7 mM hydrogen peroxide or 5 x 10^5^ F*. graminearum* conidia were performed.

### 2.9 Extraction of peroxidase substrate from *F. graminearum* conidia and its conversion into *Fg*Ste2 ligand with HRP

Freshly harvested *F. graminearum* conidia were adjusted to a concentration of 2 x 10^7^ conidia/mL. One mL of this suspension was centrifuged at 1100 g for 10 min at room temperature and resuspended in 50 µL of an aqueous solution containing HRP ranging in concentration from 4 to 120 µM, according to the experiment that was subsequently performed with the reaction product. Lower concentrations were used for initial experiments while higher concentrations of HRP were used in ligand extraction and characterization assays. Following this incubation, the mixture was centrifuged at 12,000 *g* for 10 min at room temperature and the supernatant was transferred to a fresh microfuge tube. The solution was then either boiled at 95°C for 10 min or treated with 60 mM SHAM to inactivate HRP and subsequently tested for its ability to induce chemotropism of wild type *F. graminearum* in the chemotropism plate assay.

### 2.10 Extraction of peroxidase substrate from *S. cerevisiae* cells and its conversion into *Fg*Ste2 ligand with HRP

*S. cerevisiae* cells, either wild-type or *Fg*Ste2-expressing, were inoculated in 10 mL of Synthetic Dropout Complete (SDC) medium (2% sucrose, 0.66% yeast nitrogen base, 200 mg/mL arginine, 200 mg/mL histidine, 200 mg/mL leucine, 0.2% yeast dropout mix lacking uracil, arginine, leucine and histidine) and incubated at 30°C overnight with shaking at 200 rpm. The following day, the culture was diluted to an O.D._600_ of 0.1 and incubated until they reached an O.D._600_ of 0.6-0.8 (mid-logarithmic phase). From this culture, approximately 2 x 10^7^ cells (O.D._600_ of 1 ~ 1.5 x 10^7^ cells) were collected by centrifugation and resuspended in 50 µL of 1 µM HRP and incubated at room temperature for 1 h. The suspension was then centrifuged at 1100 g for 10 min at room temperature, following which the supernatant was transferred to a fresh microfuge tube and inactivated with 10 µL of 200 mM SHAM. The extracts were then tested for chemotropic activity in wild type *F. graminearum* conidia.

### 2.11 Treatment of *F. graminearum* with glycosidases

Freshly harvested *F. graminearum* conidia were quantified with a hemocytometer. Glycosidic enzymes were kindly provided by Dr. Chantelle Capicciotti (Queen’s University, Kingston, Canada). Subsequently, 2 x 10^7^ conidia were transferred to a fresh microfuge tube and resuspended in 50 µL of glycobuffer (5 mM CaCl_2_, 50 mM sodium acetate, pH 5.5) containing either 3.75 µg or 7.5 µg of PNGase F (from *Flavobacterium meningosepticum*, NCBI accession sequence J05449), 100 µg of β-galactosidase (from *Streptococcus pneumoniae*, NCBI accession sequence ACB89855) or 100 µg of sialidase (from *Clostridium perfringens*, NCBI accession sequence WP_118429947.1). The reactions were then incubated at 37°C for 1 h, following which the samples were centrifuged at 1100 g for 10 min and then supernatant removed. The conidia were then washed twice with 1 mL of sterile deionized water. Next, 40 µL of 42 µM HRP was added to each pelleted conidia sample and incubated at room temperature for 1 h, following which the sample was centrifuged at 1100 g and the supernatant was transferred to a fresh microfuge tube. Ten microlitres of 200 mM SHAM was added to inactivate the HRP in the solution, after which it was pipetted into the test well of the chemotropism plate assay to assess chemotropic inducing potential in wild-type *F. graminearum* conidia.

### 2.12 Enrichment of ligand through liquid-liquid extraction

HRP-derived ligand was produced by treating 2 x 10^7^ spores of wild-type *F. graminearum*, in 100 µL of 4 µM HRP in a microfuge tube for 30 min at room temperature. Following this, an equal volume of 100% ethyl acetate was added. The mixture was vortexed for 30 sec and then centrifuged at 13,000 *g* for 1 min at room temperature. The ethyl acetate fraction was transferred to a fresh microfuge tube. This step was repeated, and the second ethyl acetate fraction was pooled with the first one. Next, an equal volume (100 µL) of 100% chloroform was added to the aqueous fraction, vortexed for 30 sec, and centrifuged at 13,000 *g* for 1 min at room temperature. The chloroform fraction was transferred to a fresh microfuge tube, and the step was repeated. The microfuge tubes containing the chloroform and ethyl acetate fractions were left open overnight to allow for solvent evaporation. The following day, 100 µL of deionized water was added and incubated at room temperature for 1 h. All fractions were tested for activity in the chemotropism plate assay against wild-type *F. graminearum*.

### 2.13 Enrichment of ligand by C-18 cartridge adsorption

For mass spectrometric analysis of the HRP-derived ligand, large molecular weight proteins and detergents were removed. Samples were first boiled for 5 min at 95°C, following which the sample was subjected to centrifugation in an Amicon centrifugal filter (with cutoff at 3 kDa) at 1100 g at room temperature. After centrifugation, the protein and other high molecular weight species were collected in the filter, while the ligand and other metabolites passed into the filtrate. The filtrate was then subjected to cleaning up using a C-18 cartridge. The C-18 cartridge was first pre-wet with 1 mL of 90% acetonitrile, and then equilibrated with 2 mL of deionized water. Following this, the sample was passed through, and the flowthrough was collected. The cartridge was then washed with 1 mL of water. Finally, any bound material was eluted from the cartridge with 1 mL of 90% acetonitrile. Fractions from each step of the cleanup were collected and tested for activity in the chemotropism plate assay. Before testing the elution fraction, the sample was left on the bench overnight to allow evaporation of the acetonitrile and resuspended in water the next day.

### 2.14 Sample preparation for mass spectrometric analysis

Freshly harvested *F. graminearum* conidia were quantified using a hemocytometer. For initial experiments analyzing HRP-treated and untreated wild-type extracts, 6.5 x 10^8^ conidia were resuspended in 1 mL of 180 µM HRP or water, respectively, and incubated at room temperature overnight. For the subsequent experiment, 1.5 x 10^8^ conidia of wild-type, *Fgste2Δ*, *Fgste3Δ* strains of *F. graminearum* and *Fg*Ste2-expressing *S. cerevisiae* were resuspended in 1 mL of 100 µM HRP overnight at room temperature. All samples were then processed in the same way for mass spectrometric analysis. First, the conidial suspensions in HRP were centrifuged at 11,000 *g* for 10 min to pellet the cells. Following this, the supernatant was transferred to a fresh microfuge tube and boiled at 95°C for 5 min. The sample was then passed through a pre-equilibrated C-18 column from which the flowthrough was collected and analyzed by mass spectrometry.

### 2.15 Mass spectrometry analysis

Mass spectrometry (MS) analyses were performed through two different injection modes, *i.e*., using Flow Injection Analysis (FIA) and liquid chromatography (LC).

The mass spectrometry detection was performed using a MicroTOF-Q mass analyzer (Bruker Daltonik GmbH, Bremen, Germany). The MS was operated in full scan mode (*m/z* 100-2500). For fragment confirmation, some tests were also done using the Multiple Reaction Monitoring (MRM) option with the auto-MS/MS mode and Argon for CID (collision-induced dissociation). Mass was calibrated from *m/z* 100 to *m/z* 2500 using the ESI-Low concentration Tuning Mix (Agilent Technologies, Inc., Santa Clara, CA, USA). Positive and negative electrospray ionization modes (ESI+ and ESI-, respectively) were used for both the FIA and liquid chromatography modes.

In the FIA approach, samples were directly injected into the mass spectrometer. For LC-MS, the samples were first eluted through a Luna Omega C18 PS column (150 × 4.6 mm, 3 μm particle size). The mobile phase consisted of a gradient of 0.2% acetic acid in water and methanol (0 to 100% MeOH over 25 min followed by 10 min at 100% MeOH) at a flow rate of 0.5 mL min^-1^. A 25 min hold at 100% aqueous was also added for column equilibration prior to each injection. Alternative gradients and mobile phases were tested to optimize peak separation, retention and sensitivity.

### 2.16 Total RNA extraction

*F. graminearum* strains were inoculated in 20 mL of Potato Dextrose Broth (PDB) for 2 days at 28°C with shaking at 160 rpm in the dark. For RNA extraction, dried mycelial mass was frozen in liquid nitrogen and homogenized in 1 mL TRIzol™ reagent (Thermo Fisher Scientific). The InviTrap^®^ Spin Universal RNA Mini Kit (Stratec molecular, Germany) was used to purify total RNA (free of genomic DNA) from TRIzol aqueous phase, as per the manufacturer’s protocol; the RNA concentration and purity was subsequently determined using a Nanodrop spectrophotometer ND-1000 (ThermoScientific). RNAseq libraries were prepared using TruSeq Stranded RNALT kit and sequenced on an Illumina HiSeq 2500 platform according to the manufacturer’s guidelines (Illumina, USA).

### 2.17 RNA sequencing and analysis

Libraries for RNA sequencing were prepared using TruSeq Stranded RNALT kit and sequenced on an Illumina HiSeq 2500 platform according to the manufacturer’s guidelines (Illumina, USA). The RNA sequencing data was analyzed as follows. Raw data was first trimmed using Trimmomatic v0.39 software (http://www.usadellab.org/cms/?page=trimmomatic) based on default quality scores that were determined by the base caller error probability level (P< 0.01). To assess the expression levels, high quality RNA sequences were aligned to the annotated *F. graminearum* RR1.36 genome using Salmon v1.2.1 (https://combine-lab.github.io/salmon/) (King et al., 2015). Differential expression analysis was performed using SARTools v1.6.4, with the DESeq2 settings and the parameters within their provided default template (Varet et al., 2016). A False Discovery Rate (FDR) (Benjamini and Hochberg, 1995) of corrected padj ≤ 0.05 was used as a threshold to identify differentially expressed genes. Gene annotation and GO enrichment analysis for *F. graminearum* was performed using the FungiDB database. (https://fungidb.org/fungidb/) (Stajich et al., 2012; Basenko et al., 2018). The data was then manually sorted and interrogated for pathogenicity- and chemotropism-related genes. The Full RNAseq data set is available at NCBI (Bioproject ID: PRJNA872394).

## 3 Results

### 3.1 A *Fg*Ste2-activating ligand originates from *F. graminearum* conidia

**Figure 1:**
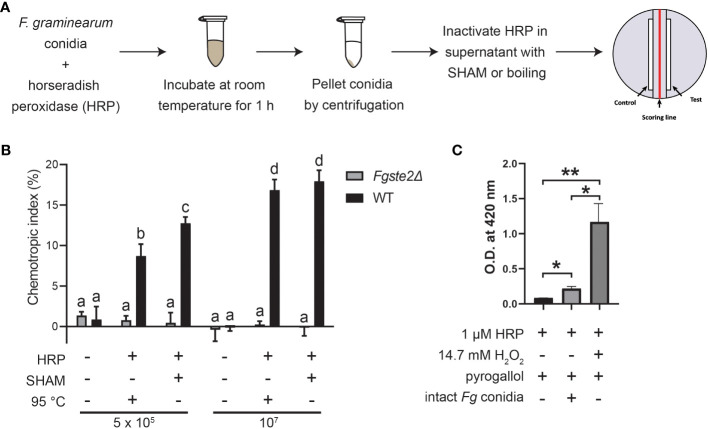
F*. graminearum* conidia produce plant peroxidase substrates that are converted to *Fg*Ste2-activating ligands causing chemotropism. **(A)** A schematic diagram depicting the general experimental approach for extracting *Fg*Ste2-activating ligand from *F. graminearum* conidia and assaying its chemotropism activity is shown. **(B)** Directed hyphal growth of wild-type and *Fgste2Δ F. graminearum* strains towards a gradient of wild-type *F. graminearum* conidial extract was measured after 14 h of exposure. Extracts were generated by treating the indicated number of wild-type *F. graminearum* conidia (either 5 x 10^5^ or 1 x 10^7^) with 4 µM HRP for 1 h at room temperature. Data were analyzed with a one-way ANOVA with multiple comparisons, using the untreated wild-type extract as a control. Bars with different letters are significantly different. b, c, d compared to a, p< 0.0001. c compared to b, p< 0.01. d compared to c, p< 0.001. Data represent the average of three independent experiments. n = 500 hyphae per plate. Error bars represent standard deviation. **(C)** A peroxidase activity assay was performed to assessing the relative levels of hydrogen peroxide secreted by *F. graminearum* conidia. Reactions contained the indicated compositions and oxidization of pyrogallol was measured by absorbance at 420 nm. Data represent the average of technical duplicates from a single experiment. Data were analyzed with a one-way ANOVA with multiple comparisons, *p< 0.05, **p< 0.01. Error bars represent standard deviation.

Previous findings showed that *Fg*Ste2 is activated by a wheat peroxidase-produced ligand, rather than the wheat-secreted peroxidase itself (Sridhar et al., 2020). To determine whether a substrate for this peroxidase reaction originates from the fungus, *F. graminearum* conidia were treated with horse radish peroxidase (HRP) in a microfuge tube, after which the conidia were pelleted, and the soluble fraction of the reaction was collected (**Figure 1A**). Any HRP in this fraction was then inactivated by the peroxidase-specific inhibitor salicylhydroxamic acid (SHAM) or by boiling for 10 min at 100°C. This solution, hereafter referred to as “extract”, induced a robust chemotropic response in wild-type *F. graminearum* in a concentration-dependent manner (**Figure 1B**). Similar responses were observed towards both the heat- and SHAM-inactivated extracts at each concentration, however, there was a slightly stronger response induced by the latter, possibly due to ligand degradation caused by boiling the samples. By comparison, negligible chemotropic growth was observed towards the extract that was not treated with HRP. The *Fgste2Δ* strain did not exhibit any chemotropic response towards the HRP-treated wild-type *F. graminearum* conidial extract, confirming that the chemotropism was *Fg*Ste2-mediated (**Figure 1B**). These results indicate that a peroxidase substrate arises from *F. graminearum* conidia and the product of the enzymatic reaction acts as a *Fg*Ste2-activating ligand.

Peroxidases catalyze the oxidation of a variety of substrates through the reduction of hydrogen peroxide, which is known to be secreted at a basal level by fungal cells (Nordzieke et al., 2019). To assess whether hydrogen peroxide is being supplied by the *F. graminearum* conidia, we monitored oxidation of pyrogallol into a colored product in a reaction containing *F. graminearum* conidia, but no added hydrogen peroxide (**Figure 1C**). In contrast to the sample containing exogenously supplied hydrogen peroxide, the *F. graminearum* conidia sample oxidized only a small amount of pyrogallol (approximately one-tenth the amount). At the same time, the conidia oxidized almost 3-fold more H_2_O_2_ than in the absence of either conidia or exogenous H_2_O_2_. Chemotropism is known to occur in response to chemical gradients of very low concentrations (Chou et al., 2011). Therefore, it is not unexpected that such a low amount of endogenous peroxide is sufficient for the production of the chemoattractant by peroxidase. Notably, a prior study with *F. oxysporum*, where they deleted the gene encoding NOX, which is the primary enzyme responsible for H_2_O_2_ generation in fungi, linked the fungal chemotropic responses to fungal derived H_2_O_2_ (Nordzieke et al., 2019) Together, these data provide further evidence that *F. graminearum* conidia provide all required peroxidase substrates.

### 3.2 *Fg*Ste2 and *Fg*Ste3 contribute to peroxidase substrate availability

**Figure 2:**
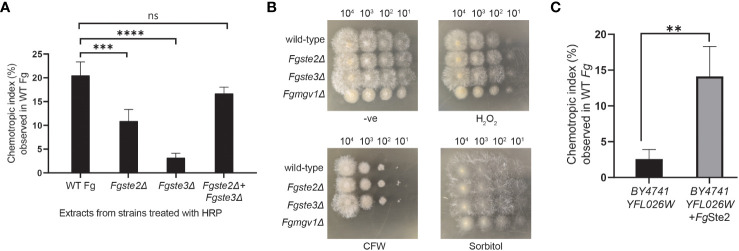
Presence of *Fg*Ste2 and *Fg*Ste3 influence ligand extraction but do not compromise *F. graminearum* resistance to growth stressors. **(A)** Directed hyphal growth of wild-type *F. graminearum* towards a gradient of peroxidase-treated extracts from wild-type, *Fgste2Δ* and *Fgste3Δ F. graminearum* conidia was measured after 14 h of exposure. Extracts were generated by treating 2 x 10^7^ conidia with 40 µM HRP for 1 h at room temperature. ***p< 0.001, ****p< 0.0001, compared to WT extract. Data represent the average of three independent experiments. n = 500 hyphae per plate. Error bars indicate standard deviation. **(B)** Serial dilutions of the indicated strains of *F. graminearum* conidia were spotted onto PDA plates containing either no stressor (-ve), cell wall integrity stressor (300 µg/mL Calcofluor White; CFW), osmotic stressor (1M Sorbitol), or oxidative stressor (0.2 mM H_2_O_2_). Plates were incubated for 48 h at 25°C and imaged. One representative image for each plate is shown. *Fgmgv1Δ* lacking MAPK in the CWI pathway was used as a control for the CFW condition. **(C)** Directed hyphal growth of wild-type *F. graminearum* towards a gradient of HRP-treated *S. cerevisiae* extracts was measured after 14 h of exposure and compared to *BY4741 YFL026W* extract, **p< 0.01. Data represent the average of three independent experiments and were analyzed with a student’s t-test. n = 500 hyphae per plate. Error bars indicate standard deviation.

To investigate the involvement of the *Fg*Ste2 and *Fg*Ste3 receptors in provision of the substrate that plant peroxidases convert into a chemotropism-inducing ligand, *Fgste2Δ* and *Fgste3Δ* conidia were suspended in a solution containing 42 μM HRP and then the soluble fraction from this reaction was assessed for its ability to induce chemotropism in wild-type *F. graminearum*. Based on our previous observations, 4μM of HRP produced a similar degree of *F. graminearum* chemotropism as peroxidase derived from wheat exudate (Sridhar et al., 2020). Thus, we reasoned that the 10-fold excess of HRP used in the current assay would ensure that peroxidase was not limiting during the treatment of each strain of *F. graminearum* conidia. **Figure 2A** shows that the HRP-treated *Fgste2Δ* conidial extract induced chemotropism of wild-type *F. graminearum* at approximately 50% that of the extract derived from wild-type conidia. Conversely, the HRP-treated extract from *Fgste3Δ* conidia induced a negligible chemotropic response. These results suggest that *Fg*Ste2 and *Fg*Ste3 are involved in the synthesis and/or presentation of the peroxidase substrate on the surface of *F. graminearum* cells.

To explore whether *Fg*Ste2 and *Fg*Ste3 regulate availability of the same substrate to different extents, or the availability of two separate substrates, a mixture of *Fgste2Δ* and *Fgste3Δ* conidia was treated with HRP to generate a mixed extract. Remarkably, the chemotropic response induced by the mixed extract was comparable to the extract from wild-type conidia. Altogether, these results suggest that *Fg*Ste2 and *Fg*Ste3 regulate the availability of two different substrates that are both chemically modified by HRP to produce an *Fg*Ste2-activating ligand.

Finally, to examine the possibility that knocking out *FgSTE2* and *FgSTE3* induced phenotypic changes that simply limit the amount of ligand being extracted by peroxidase treatment of the conidia, rather than the amount of substrate produced by the cells, a dilution spot assay was performed to assess the growth of *Fgste2Δ* and *Fgste3Δ* on media plates containing a panel of *F. graminearum* growth stressors (**Figure 2B**). Neither strain showed defects in any of the conditions tested, however, *Fgste2Δ* and *Fgste3Δ* produced colonies with longer hyphae on hydrogen peroxide-containing plates compared to wild type. This result indicates a role for *Fg*Ste2 and *Fg*Ste3 in oxidative stress response. Together, these data indicate that reduction of chemotropic ligand generated by peroxidase treatment of these mutant strains is likely due to downregulation and/or reduced presentation of peroxidase substrates on the cell surface when *Fg*Ste2 and *Fg*Ste3 are absent.

### 3.3 Expression of *Fg*Ste2 in *Saccharomyces cerevisiae* induces availability of a peroxidase substrate that is converted to an *Fg*Ste2-activating ligand

To determine whether expression of *Fg*Ste2 in *S. cerevisiae* could induce availability of a peroxidase substrate, the *FgSTE2* gene was cloned into the pYES-DEST52 plasmid. This plasmid adds a C-terminal 6x-His tag to *Fg*Ste2 and allows control of its expression via a galactose-inducible promoter. Successful integration of the *FgSTE2* gene into the plasmid was verified by Sanger sequencing and the DNA was subsequently transformed into a strain of *S. cerevisiae* lacking the endogenous *ScSTE2* (*BY4741 YFL026W*) by electroporation (**Supplementary Figure S1A**). Transformants that grew on selective media lacking uracil were verified by amplification of the *FgSTE2* gene by polymerase chain reaction (**Supplementary Figure S1B**). Expression of *Fg*Ste2 was then assessed by Western Blotting using an anti-His antibody (**Supplementary Figure S1C**). As expected, *Fg*Ste2 was not expressed when cells were cultured in YPD, due to suppression of the galactose-inducible promoter by glucose, while expression of *Fg*Ste2 was induced when galactose was provided in the growth media (**Supplementary Figure S1C**).

To test for production of peroxidase substrate, *Fg*Ste2-expressing *S. cerevisiae* cells were harvested and treated with HRP. Remarkably, the *Fg*Ste2-expressing *S. cerevisiae* extract induced a robust chemotropic response in wild-type *F. graminearum* (**Figure 2C**). By contrast, an extract from *S. cerevisiae* cells lacking *Fg*Ste2 or *Sc*Ste2 did not induce a significant chemotropic response. Thus, *Fg*Ste2 enhances availability of a peroxidase substrate in *S. cerevisiae* that can be converted to a *Fg*Ste2-activating ligand that stimulates *F. graminearum* chemotropism. The increased peroxidase substrate availability when *Fg*Ste2 is expressed in *S. cerevisiae* cells may not be comparable to its reduction when *Fg*Ste2 is deleted in *F. graminearum*. In *S. cerevisiae*, *Fg*Ste2 is overexpressed by the strong galactose-inducible promoter which likely enhances any impact of *Fg*Ste2 on peroxidase substrate availability.

### 3.4 The peroxidase-derived *Fg*Ste2 ligand is a polar molecule

**Figure 3:**
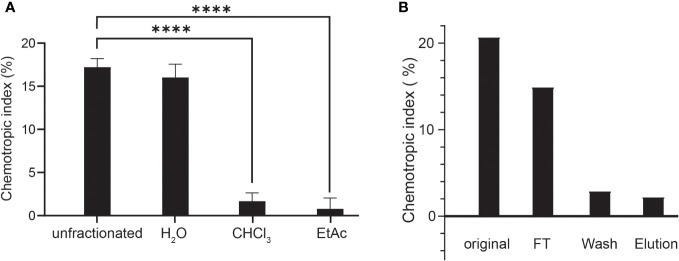
The peroxidase-generated *Fg*Ste2-activating ligand is hydrophilic and polar. **(A)** Directed hyphal growth of wild-type *F. graminearum* towards fractions collected after liquid-liquid fractionation of the peroxidase-treated wild-type *F. graminearum* conidial extract was measured after 14 h of exposure and compared to the unfractionated control (****p< 0.0001). Data represent the average of three independent experiments and were analyzed with a one-way ANOVA. n = 500 hyphae per plate. Error bars indicate standard deviation. H_2_O, aqueous; CHCl_3_, chloroform; EtAc, Ethyl acetate. **(B)** HRP-treated wild-type conidial extract was fractionated by adsorption using a C-18 cartridge and the flowthrough (FT), wash and elution were collected. Directed hyphal growth of wild-type *F. graminearum* towards purification fractions was measured after 14 h of exposure. Data is representative of one experiment. n = 500 hyphae per plate.

To isolate and characterize the *Fg*Ste2-activating ligand produced by plant peroxidase, liquid-liquid extraction was used to separate components of the HRP-treated *F. graminearum* conidial extract based on their solubilities (**Figure 3A**). Each fraction was then tested for induction of chemotropism in wild-type *F. graminearum*. The aqueous fraction, but not the chloroform or ethyl acetate fractions, induced a robust chemotropic response (**Figure 3A**), indicating that the peroxidase-generated *Fg*Ste2 ligand is a highly polar molecule. To facilitate detection by mass spectrometry, a C-18 cartridge was used to selectively remove any components in the conidial extract that may interfere with the analysis. The ligand did not bind to the C-18 cartridge and eluted in the flowthrough fraction, again indicative of a highly polar molecule (**Figure 3B**). The flowthrough from this step was analyzed by mass spectrometry.

### 3.5 Characterization of the *Fg*Ste2 ligand by mass spectrometry

**Figure 4:**
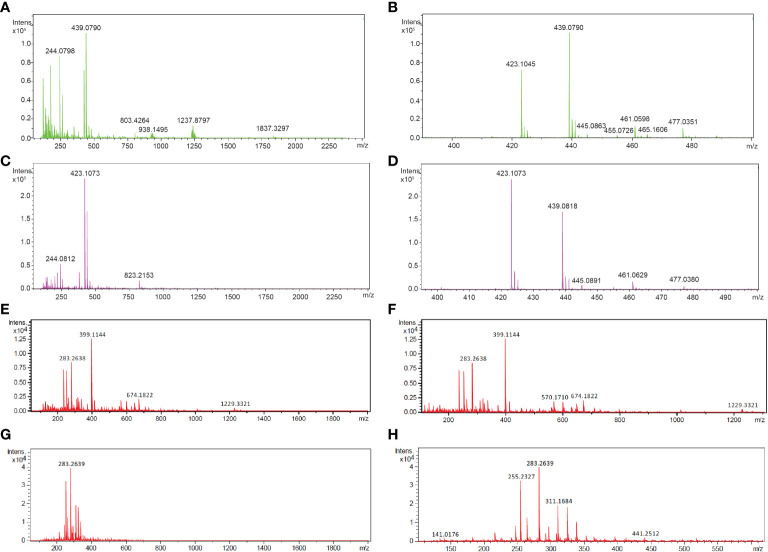
Mass spectra of HRP-treated and untreated wild-type *F. graminearum* conidial extracts. **(A)** Full (50 to 2500 *m/z*) and **(B)** zoomed in (390 to 500 *m/z*) mass spectra, acquired in positive ionization (ESI+) mode after flow injection are shown for undiluted HRP-treated extract with the masses of the most abundant peaks labelled in with their *m/z*. **(C)** Full (50 to 2500 *m/z*) and **(D)** zoomed in (390 to 500 *m/z*) mass spectra, acquired in positive ionization (ESI+) mode after flow injection is shown for the untreated extract with the masses of the most abundant peaks labelled in with their *m/z*. **(E)** Full (50 to 2000 *m/z*) and **(F)** zoomed in (100 to 1300 *m/z*) mass spectra, acquired in negative ionization (ESI-) mode after flow injection is shown for the 3-fold diluted HRP-treated extract with the masses of the most abundant peaks labelled in *m/z*. **(G)** Full (50 to 2000 *m/z*) and **(H)** zoomed in (100 to 1300 *m/z*) mass spectra, acquired in negative ionization (ESI-) mode after flow injection is shown for the 3-fold diluted untreated extract with the masses of the most abundant peaks labelled in *m/z*. Collision energy was set at 10 V for all analyses.

To determine the composition of the *Fg*Ste2 ligand that derived from peroxidase treatment of *F. graminearum* conidia, Flow Injection Analysis (FIA) was applied to the flowthrough material from C-18 adsorption. Several unique masses were identified by FIA at a higher intensity in the HRP-treated extract compared to the untreated control under both positive and negative modes of ionization (**Figure 4**). With the positive mode of ionization, notable masses at 423 *m/z* and 439 *m/z* were identified in the HRP-treated extract (**Figures 4A, B**) and the untreated extract (**Figures 4C, D**). In negative ionization mode however, one prominent mass was observed at 399 *m/z* in the HRP-treated extract (**Figures 4E, F**), which was absent in the untreated extract (**Figures 4G, H**).

**Figure 5:**
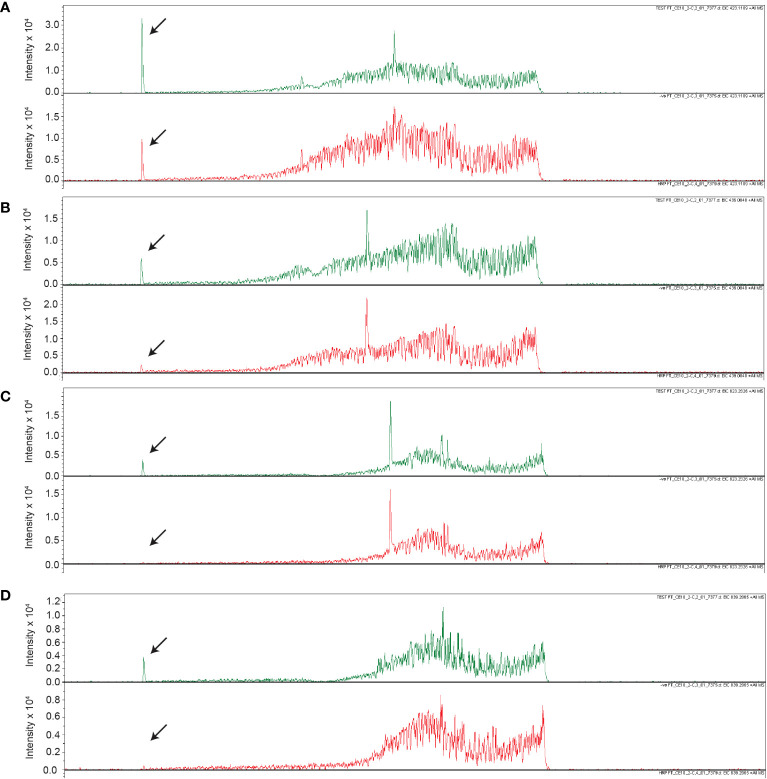
Unique masses in HRP-treated extract are present in a higher abundance than the untreated control. Extracted ion chromatograms obtained from LC-MS under positive ionization (ESI+) of HRP-treated extracts are shown for **(A)** 423 *m/z*, **(B)** 439 *m/z*, **(C)** 823 *m/z*, **(D)** 839 *m/z*. Chromatograms for HRP-treated and untreated extracts are shown in green and red, respectively.

Upon further investigation of treated and untreated samples using LC-MS with a Luna Omega C18 PS column, a series of related peaks were detected corresponding to masses 418, 423, 439, 818, 823 and 839 Da, that all co-eluted at 6.6 min (**Figure 5**). These masses were detected in positive mode of ionization. For each of these masses, the intensity of the peak was approximately 80% lower in the untreated control compared to the HRP-treated sample (**Figure 5**). Interestingly, the masses in the *m/z* 400 range differed from those in the 800 *m/z* range by a constant 400 Da. A less steep elution gradient (increase of 1% per min compared to 4% per min used earlier), as well as other columns and mobile phases, were used in an attempt to resolve peaks potentially belonging to different compounds. No difference in elution was observed, suggesting that all masses originated from, or were adducts of, a common parent compound.

The *m/z* 418 mass was determined to have an exact molecular weight of 418.1565 Da, matching the expected mass of an ammonium adduct of the 400 Da species (theoretical exact mass: 418.1555 Da). Similarly, the 423 and 438 Da masses were confirmed to be Na^+^ and K^+^ adducts of the 400 Da species (**Table 4**). In addition to the adducts of this 400 Da compound, dimers and trimers were also detected in the sample. Based on the measured mass of the pseudomolecular ion [M-H]^-^ (measured mass: 399.1143 Da; theoretical exact mass: 399.1144 Da; mass error: 0.25 mDa), a molecular formula of C_14_H_24_O_13_ was proposed for the 400 Da compound, indicating a high level of oxygenation with almost one oxygen per carbon atom present. This is suggestive of a disaccharide molecule.

**Table 4:** Summary of pseudomolecular ions or adducts of the compound eluting at 6.6 min with the Luna Omega C18 PS column.

Ionization mode	Measured *m/z*	Ions proposed	Chemical formula of ion	Calculated exact mass *m/z*
ESI+	401.1298	[M+H]^+^	C_14_H_25_O_13_	401.1290
	383.1204	[M+H-H_2_O]^+^	C_14_H_23_O_12_	383.1184
	418.1565	[M+NH_4_]^+^	C_14_H_28_NO_13_	418.1555
	423.1121	[M+Na]^+^	C_14_H_24_NaO_13_	423.1109
	439.0845	[M+K]^+^	C_14_H_24_KO_13_	439.0848
	823.2328	[2M+Na]^+^	C_28_H_48_NaO_26_	823.2326
	839.2027	[2M+K]^+^	C_28_H_48_KO_26_	839.2065
ESI-	399.1143	[M-H]^-^	C_14_H_23_O_13_	399.1144
	799.2321	[2M-H]^-^	C_28_H_47_O_26_	799.2361

**Figure 6:**
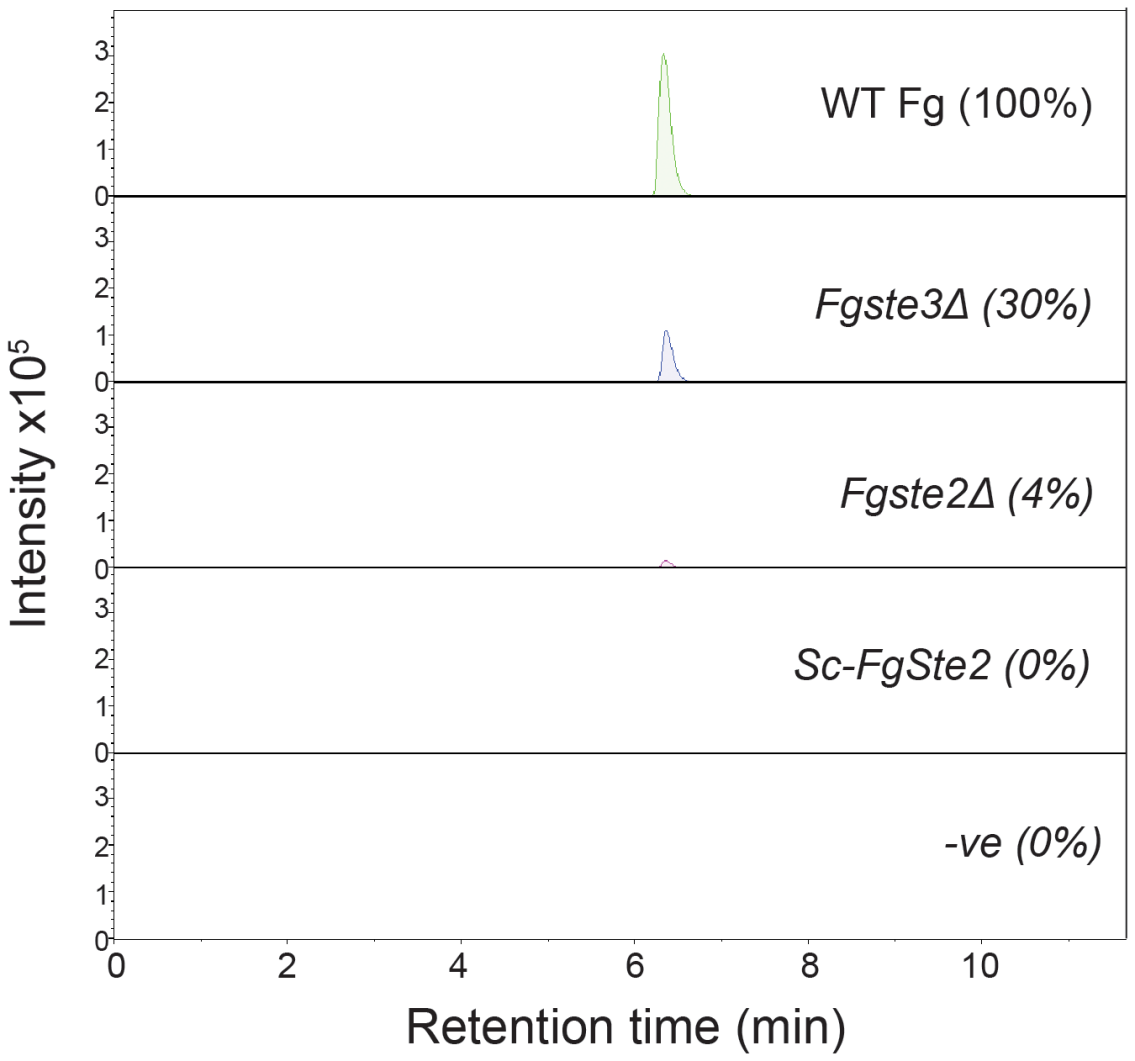
Comparison of *m/z* 399 abundance in extracts from different strains. Extracted ion chromatograms for the *m/z* 399 species detected under ESI- conditions are shown for extracts from wild-type (WT), *Fgste3Δ*, *Fgste2Δ*, *Fg*Ste2-expressing *S. cerevisiae* (*Sc-FgSte2*) and HRP solution (-ve). Relative abundances (measured by area under the peak) of *m/z* 399 mass from each extract are included in parentheses. Intensities were measured relative to the wild-type (WT) extract.

The intensity of the 400 Da peak was 70% and 96% lower in HRP-treated *Fgste3Δ* and *Fgste2Δ* conidial extracts, respectively, compared to the wild-type *F. graminearum* extract (**Figure 6**). Diminished abundance of the 400 Da peak in the *Fgste3Δ* extract likely accounts for the weak chemotropic response it induced in wild-type *F. graminearum* conidia (**Figure 2A**). However, the lack of 400 Da species in the HRP-treated *Fgste2Δ* extract does not correlate with the moderate chemotropic response induced by this extract. Thus, there may be a second ligand component that is not regulated by *Fg*Ste2, and the mass of which is not detected by the present mass spectrometric analysis.

The 400 Da species was also absent in the extract from *Fg*Ste2-expressing *S. cerevisiae* cells (**Figure 6**). Any differences between the extracts generated from *Fg*Ste2-expressing *F. graminearum* and *S. cerevisiae* cells could be due to the induction and/or presentation of different compounds that are both capable of stimulating chemotropic growth in wild-type *F. graminearum*.

### 3.6 The *Fg*Ste2-activating ligand is likely a carbohydrate

**Figure 7:**
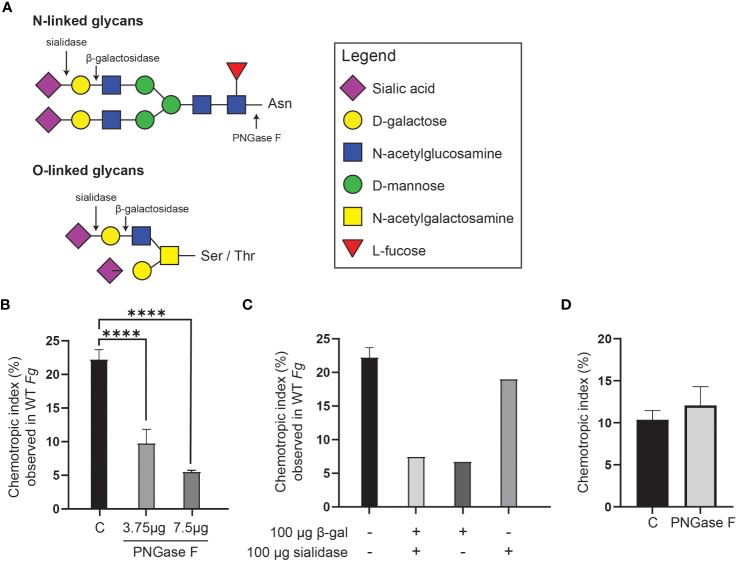
Glycosidase treatment of *F*. *graminearum* conidia leads to decrease in extraction of *Fg*Ste2-activating ligand by peroxidase. **(A)** A schematic representation of the general structures of N- and O-linked glycosylation of proteins is shown. Cleavage sites for PNGase F (Cγ-Nδ bond between-acetylglucosamine and Asparagine residue), β-galactosidase (β-1,4-D-galactosidic bond), and sialidase (α2-3, α2-6, and α2-8 linked sialic acid residues) are shown. **(B)** Wild-type *F*. *graminearum* conidia were pretreated with indicated amounts of PNGase F (3.75 µg or 7.5 µg) or not treated with PNGase F **(C)**. HRP-treated extracts were then generated from these conidia and assessed for chemotropism in wild-type *F. graminearum* conidia. Data represent the average of three independent experiments and were analyzed with a one-way ANOVA. n = 500 hyphae per plate. Error bars indicate standard deviation. **(C)** Wild-type *F. graminearum* conidia were pretreated with indicated combinations of β-galactosidase and sialidase. HRP-treated extracts were then generated from these conidia and assessed for chemotropism in wild-type *F. graminearum* conidia. Graph represents data from a single experiment. n = 500 hyphae per plate. **(D)** Directed hyphal growth of wild-type *F*. *graminearum* towards a gradient of 4 µM HRP, either untreated **(C)** or pretreated with PNGase F (PNGase F). Data represent the average of three independent experiments. n = 500 hyphae per plate. Error bars represent standard deviation. ****p < 0.0001.

On the basis that the 400 Da species has a molecular formula compatible with a disaccharide, and that peroxidases can promote degradation of carbohydrates (Schweikert et al., 2002), *F. graminearum* conidia were pre-treated with three glycosidases, PNGase F, sialidase, and β-galactosidase either individually or in combination, followed by a washing step, prior to HRP treatment and chemotropism assessment. (**Figure 7**). Each of these enzymes cleaves at specific glycosidic bonds within a carbohydrate chain (**Figure 7A**).

Pre-treatment with PNGase F significantly lowered the chemotropic response to the peroxidase-treated extract compared to the control (C) extract that was not exposed to glycosidase (**Figure 7B**). A combination of β-galactosidase and sialidase also resulted in an extract that induced a lower chemotropic response compared to the control, suggesting that a reduced amount of chemotropic ligand was present (**Figure 7C**). When the glycosidases were applied individually, it was observed that the β-galactosidase treatment, and not the sialidase, limited chemotropism the most (**Figure 7C**). These observations indicate that the ligand is most likely a carbohydrate or its derivative. That treatment of *F. graminearum* conidia with the glycosidases did not completely eliminate a chemotropic response (about 50% compared to the control) could be due to limited access to some glycosidic bonds in the conidia or reduced activity of the purified glycosidases. Alternatively, the second peroxidase substrate may be present on a different cell wall or membrane component and not associated with glycans and therefore not cleaved off by glycosidase treatment.

Although reports in the literature show that PNGase F does not cleave the glycans on HRP (Yang et al., 1996; Wang et al., 2014), an independent experiment was performed where a mixture of HRP and PNGase F was tested as a stimulus in the chemotropism plate assay to ensure that the HRP was not inactivated by PNGase F. As expected, no decrease in chemotropism was observed compared to the control lacking PNGase F (**Figure 7D**). This further demonstrates that the loss of chemotropic response by the glycosidase-treated conidial extracts was likely due to a reduction in the amount of peroxidase substrate available for conversion to the *Fg*Ste2-activating ligand.

### 3.7 Loss of *Fg*Ste2 in *F. graminearum* affects expression of cell wall and cell membrane-related genes

The reduced amount of *Fg*Ste2-activating ligand that can be extracted from *Fgste2Δ* conidia indicates that the presence of the *Fg*Ste2 receptor is important for availability of the peroxidase substrate, either through regulation of its synthesis and/or its display on the cell surface. In addition, the presence of this peroxidase substrate would have to be independent of the activation status of *Fg*Ste2 being that the receptor is not being activated until after the substrate is available. Transcriptomic analyses were therefore performed on wild type and *Fgste2Δ* cells to gain a deeper understanding of the cellular and metabolic processes that may be regulated by *Fg*Ste2. Genes that were upregulated in *Fgste2Δ* were not considered in order to simplify identification of FgSte2-dependent gene expression networks.

**Figure 8:**
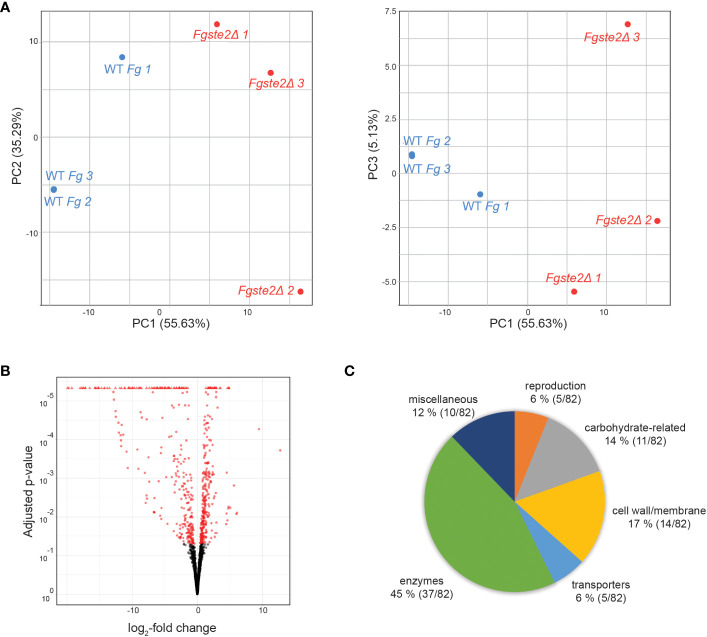
Overall distribution of genes whose expression level is altered by loss of FgSte2 in *F. graminearum*. **(A)** Principal Component Analysis (PCA) and **(B)** Volcano plot of differential gene expression in *Fgste2Δ* relative to wild-type *F. graminearum*. Plots were obtained using DESeq2 software. **(C)** Genes downregulated in *F*. *graminearum* when *FgSTE2* was knocked out were categorized based on biological function and represented as a pie chart. The percentage of genes in each biological function and the fraction of those genes among the total downregulated are indicated.

A total of 155 genes were differentially expressed when *FgSTE2* was deleted from *F. graminearum* (padj< 0.05, -1.0 > log2FC > 1.0); 22 genes were upregulated (log2FC > 1.0) (**Supplementary File 2**, **Supplementary Table S1**) and 133 genes were downregulated (log2FC< -1.0) (**Figures 8A, B**, **Supplementary File 2**, **Supplementary Table S2**). Of the downregulated genes, 51 genes had no predicted function and are listed as “hypothetical proteins” or “unnamed protein product”. The remaining 82 genes had varying biological functions according to FungiDB annotations and BLASTp predictions (**Figure 8C**). 17% of the downregulated genes have cell wall and cell membrane-related functions (**Table 5**), of which several are post-translationally modified with carbohydrate moieties. Among these were genes predicted to encode cell wall mannoprotein Cis3 (Ghanegolmohammadi et al., 2021), surface protein Sp1, mannose-specific lectin, spore coat protein sp96 precursor (Seong et al., 2008) and a cysteine-rich cell wall protein. Genes for integral membrane proteins, another putative surface protein Sp1, putative cell wall protein Sed1 (Lee et al., 2010), and an uncharacterized cell wall glycoprotein also exhibited diminished expression levels when *FgSTE2* was absent.

**Table 5:** Downregulated cell wall- and carbohydrate-related genes in *Fgste2Δ* compared to wild-type *F. graminearum*.

Function	Gene ID	Putative function	Log_2_FC	padj
Cell wall-related	*FGRAMPH1_01G05235*	Cis3 cell wall mannoprotein	-4.599	1.71E-06
	*FGRAMPH1_01G12689*	Sp1 surface protein	-2.506	0.022153
	*FGRAMPH1_01G21895*	Mannose-specific lectin	-3.565	0.000248
	*FGRAMPH1_01G13831*	Spore coat precursor Sp96	-6.252	0.000129
	*FGRAMPH1_01G11517*	Cysteine-rich cell wall protein	-8.321	0.000738
	*FGRAMPH1_01G25189*	Integral membrane proteins	-1.477	0.014578
	*FGRAMPH1_01G25309*		-3.554	0.00406
	*FGRAMPH1_01G13321*		-6.119	0.000352
	*FGRAMPH1_01G13163*		-2.36	2.07E-05
	*FGRAMPH1_01G25175*		-6.79	1.82E-08
	*FGRAMPH1_01G21921*	Sp1 surface protein	-6.634	6.67E-06
	*FGRAMPH1_01G19815*	Sed1	-5.438	2.94E-15
	*FGRAMPH1_01G11715*	Cell wall glycoprotein	-7.101	3.52565E-07
Carbohydrate-related	*FGRAMPH1_01G08099*	Wall Stress-responsive Component-containing (WSC) proteins	-5.358	2.25E-10
	*FGRAMPH1_01G18707*		-3.115	6.89E-10
	*FGRAMPH1_01G24315*		-4.45	9.52E-14
	*FGRAMPH1_01G25869*		-4.68	0.000383
	*FGRAMPH1_01G18323*	Chitin synthase IV	-4.671	0.000492
	*FGRAMPH1_01G02775*	Endochitinase I	-5.04	8.65E-11
	*FGRAMPH1_01G26041*	Probable family 17 glucosidase	-2.248	0.000278
	*FGRAMPH1_01G10193*	Beta-1,3-glucan-binding protein	-2.872	4.5E-09
	*FGRAMPH1_01G10193*	Endo alpha polygalactosaminidase precursor	-2.872	4.5E-09
	*FGRAMPH1_01G20841*	N-acetylglucosaminyltransferase I	-6.753	4.12E-07
	*FGRAMPH1_01G10191*	Family 2 glycosyltransferase	-3.165	1.3E-06

Interestingly, deletion of *FgSTE2* also led to the downregulation of several carbohydrate-related genes. A BLASTp search of four genes encoding hypothetical proteins identified the presence of Wall Stress-responsive Component (WSC) domain. In *S. cerevisiae*, WSC domain-containing proteins have been implicated in carbohydrate binding and are also proposed to act as cell wall sensors for the cell wall integrity MAPK pathway (Verna et al., 1997; Lodder et al., 1999). Proteins containing this domain have been identified as having roles in osmotic and acidic pH stress tolerance (Futagami et al., 2011) and methanol sensing (Ohsawa et al., 2017) in other fungal species. Furthermore, genes encoding the enzymes chitin synthase IV (Martín-Urdíroz et al., 2008), endochitinase I, and family 17 glycoside hydrolase may contribute towards the polymerization and degradation of fungal cell wall, suggesting that they are important in cell wall remodeling. The gene *FGRAMPH1_01G10193* is predicted to encode a beta-1,3-glucan-binding protein, though its function remains unclear. *FGRAMPH1_01G21439* is predicted to encode an endo alpha polygalactosaminidase precursor that degrades polysaccharides in the plant cell wall in *Fusarium virguliforme* (Chang et al., 2016). In addition, the N-acetylglucosaminyltransferase I-encoding gene, *FGRAMPH1_01G20841*, is downregulated upon deletion of *FgSTE2*, and a family 2 glycosyltransferase implicated in facilitating pathogenicity by enabling hyphal growth on solid surfaces (King et al., 2017) was also downregulated.

Apart from the cell wall and carbohydrate-related genes, several others were downregulated by knockout of *FgSTE2* that may have overall biological relevance (**Table 6**). This includes several reproduction-related genes, such as *abaA*, *brlA* and *wetA*. These genes act both individually and in concert to regulate their own expression as well as that of numerous other sporulation-specific genes in *F. graminearum* (Son et al., 2013; Son et al., 2014) and *Aspergillus nidulans* (Boylan et al., 1987; Mirabito et al., 1989; Sewall et al., 1990; Sewall, 1994). This is consistent with Ste2’s role as a pheromone receptor and its involvement in sexual reproduction. In addition, a gene encoding the heterokaryon incompatibility protein was downregulated. Heterokaryon incompatibility proteins are essential for preventing the fusion of non-compatible hyphae and nuclei (containing different alleles at the het locus) during hyphal fusion and branching (Saupe, 2000; Glass and Kaneko, 2003). Overall, *Fg*Ste2 has a role in reproduction, consistent with its pheromone sensing function, and may also constitutively regulate the cell wall integrity pathway.

**Table 6:** Other biologically relevant genes downregulated in *Fgste2Δ* compared to wild-type *F. graminearum*.

Function	Gene ID	Putative function	Log_2_FC	padj
Reproduction-related	*FGRAMPH1_01G02219*	abaA	-5.247	9.66E-20
	*FGRAMPH1_01G03843*	brlA	-1.844	3.86E-06
	*FGRAMPH1_01G07441*	wetA	-5.13	9.07E-08
	*FGRAMPH1_01G25435*	Heterokaryon incompatibility protein	-2.148	0.000784

## 4 Discussion

Since Ste2 was first identified as a pheromone-sensing GPCR in *S. cerevisiae* (Hartwell, 1980), extensive effort has been devoted to understanding its activation mechanism and the downstream effects thereof. The ease of genetic manipulation and high throughput assays of *S. cerevisiae* have enabled the dissection of some of these elements of *Sc*Ste2p. While its best studied function is in mating where it binds the α-pheromone peptide and leads to a well-defined set of cellular responses mediated by the heterotrimeric G protein (Schrick et al., 1997), *Sc*Ste2p can be activated by higher concentrations of α-pheromone and lead to G protein-independent signaling through α-arrestins (Choudhary and Loewen, 2016b). Other functions for this receptor have been determined as well (Jackson et al., 1991; Segall, 1993; Brizzio et al., 1996; Elia, 1996; Barkai et al., 1998; Dube and Konopka, 1998; Shi et al., 2007; Choudhary and Loewen, 2016a), however, these have primarily been in relation to varying concentrations of α-pheromone peptide or mutations generated in the receptor leading to differences in signaling. More recently, several studies have established a role for Ste2 in pathogenicity in more complex fungi (Turrà et al., 2015; Sridhar et al., 2020; Vangalis et al., 2022), where Ste2 is responsible for sensing and mediating chemotropism towards host-secreted compounds. In these organisms, Ste2 is also responsible for responding to α-pheromone peptides, demonstrating its ability to recognize multiple ligands.

In investigating the source of the substrate that plant peroxidases convert into a *Fg*Ste2-activating ligand, we found that *F. graminearum* conidia are a key producer of this material and that the hydrogen peroxide required for catalysis of the reaction is generated by the fungus as well. Class III peroxidases catalyze substrate conversion through two main mechanisms. First, they simultaneously oxidize and reduce phenolic monomers and hydrogen peroxide, respectively, which facilitates remodeling and reinforcement of the plant cell wall (Passardi et al., 2004; Almagro et al., 2009; Cosio and Dunand, 2009). Second, they convert hydrogen peroxide into hydroxyl radicals in the presence of NADH, which can attack and degrade polysaccharide chains, also important for plant cell wall remodeling (Schweikert et al., 2002). Release of the *Fg*Ste2-activating ligand after peroxidase modification of a *F. graminearum* conidial component is more reminiscent of the degradation and release of polysaccharides by the peroxidase than peroxidase-mediated polymerization of phenolic compounds.

In *F. graminearum*, the cell membrane and cell wall represent abundant and accessible reservoirs for carbohydrate and phenolic compounds. The fungal cell wall is an intricate structure composed of dense layers of polysaccharides, glycoproteins and other metabolites and pigments. Although the cell wall in *S. cerevisiae* is well studied, less is known about their counterparts in filamentous fungi. In *F. graminearum*, carbohydrates account for almost 75% of biomolecules present in the cell wall (Barbosa and Kemmelmeier, 1993), thus making it a probable source of a sugar substrate. The cell wall of *F. graminearum* also contains pigments, some of which are aromatic or phenolic, that afford this pathogen resistance to radiation and other microbes as well as enhanced pathogenicity on its hosts (Cambaza, 2018). These polysaccharide and metabolite pools represent a possible source for the peroxidase substrate.

Purification of the *Fg*Ste2-activating ligand through liquid-liquid extraction and C-18 cartridge-based adsorption suggest that it is a polar compound. Mass spectrometry analyses supported this result and revealed the presence of a highly oxygenated species of 400 Da that was absent or reduced in the negative controls. Based on its elution time from the C-18 column and its molecular formula (C_14_H_24_O_13_), the 400 Da compound appears to be a disaccharide or its derivative. Indeed, sugar-activated GPCRs have been identified in other systems, including Gpr1p in *S. cerevisiae* (Lemaire et al., 2004), and GPR35 (Foata et al., 2020) and sweet taste receptors in mammals (Li et al., 2002). Peroxidase treatment of wild-type *F. graminearum* conidia whose glycans had been previously cleaved off resulted in an extract that induced a lower chemotropic response. This further validates the identity of the *Fg*Ste2-activating ligand as a carbohydrate and suggests that it may be derived from a glycan chain of an N-glycosylated protein, several of which are known to be anchored to different parts of the cell membrane and cell wall.

The discovery that loss of *Fg*Ste2 and *Fg*Ste3 resulted in extraction of lower amounts of ligand was unexpected and implies that there is a direct link between receptor expression/presence and peroxidase substrate synthesis and/or its cell surface display. Moreover, an extract generated from treating a mixture of *Fgste2Δ* and *Fgste3Δ* conidia with peroxidase induces a chemotropic response equivalent to that of wild type, suggesting that *Fg*Ste2 and *Fg*Ste3 each regulate or present a different peroxidase substrate.

These data also suggest that *Fg*Ste2 and *Fg*Ste3 exhibit basal activity (i.e., signaling in the absence of a ligand), resulting in constitutive regulation of certain genes that are involved in synthesis or display of the peroxidase substrate. Constitutive or basal signaling by GPCRs has been reported for several mammalian receptors (Nakashima et al., 2013; Meye et al., 2014; Lamichhane et al., 2015; Krumm et al., 2016; Wilde et al., 2016; Lu et al., 2021). While it has not been reported for the wild-type *Sc*Ste2p receptor, certain mutations in transmembrane helix 6 confer constitutive activity (Choudhary and Loewen, 2016a). The pheromone-like gene *CPR2* in *Cryptococcus neoformans* was discovered to encode a GPCR that was able to constitutively activate pheromone responses even in the absence of the mating factor (Hsueh et al., 2009). Beyond this, evidence for constitutively active fungal GPCRs in the literature is sparse. RNA sequencing revealed that Ste2 in *F. graminearum* appears to be responsible for regulating several carbohydrate-related enzymes that may have relevance for synthesis and assembly of carbohydrate moieties, and their conjugation to other biomolecules, that could become plant peroxidase substrates. Furthermore, deletion of *FgSTE2* leads to downregulation of several elements at different levels of the cell wall integrity MAPK pathway, suggesting that it may constitutively activate this pathway.

The data obtained from RNA sequencing is interesting in light of the mass spectrometry results. Despite the initial detection of multiple masses with the same retention time, these were all confirmed to be adducts of a single unique compound. The mass spectrometry and chemotropism data for the *Fgste3Δ*-derived extract and clearly link *Fg*Ste3 to the production and/or display of the 400 Da species. However, the mass spectrometry data also links *Fg*Ste2 to the regulation and/or display of the 400 Da species, where its deletion eliminated almost all accumulation of the 400 Da species. Furthermore, this *Fgste2Δ* extract (lacking any 400 Da species) still induces a moderate chemotropic response in wild-type *F. graminearum*, consistent with a second peroxidase substrate being present and likely linked to *Fg*Ste3. However, a different method of isolation or detection may be required to identify this second molecule.

The 400 Da mass was not detected in the extract generated from peroxidase treatment of *Fg*Ste2-expressing *S. cerevisiae* cells. It is therefore likely that *Fg*Ste2 is responsible for the synthesis or display of a different peroxidase substrate in the *S. cerevisiae* system. It is probable that the impact of *Fg*Ste2 on each system differs due to the unique biology of *F. graminearum* and *S. cerevisiae*.

In conclusion, we have demonstrated that wheat secreted peroxidases convert a substrate present on *F. graminearum* conidia into a ligand that activates chemotropism in the fungus through the *Fg*Ste2 and *Fg*Ste3 receptors. Based on the data presented in this study, *Fg*Ste2 and *Fg*Ste3 influence the availability of this substrate, which appears to be a carbohydrate. With this data, we can begin to build a model of the possible molecular mechanisms underlying host-directed chemotropism in *F. graminearum*. *Fg*Ste2 and *Fg*Ste3 both appear to regulate the synthesis or cell surface display of a 400 Da carbohydrate moiety. Carbohydrates can be found in various forms at different locations of the cell. They are involved in metabolic processes such as glycolysis or are conjugated to other biomolecules in processes like glycosylation (Deshpande et al., 2008). Some of these may be trafficked to the exterior of the cell, such as on glycosylated proteins or secreted from the cell. In addition, *Fg*Ste3 may regulate the availability of a second peroxidase substrate that was not detected by mass spectrometry, although this remains to be validated. We propose that when these substrates are exposed to peroxidases secreted from the wheat plant they are chemically modified and cleaved off their anchor in the cell membrane or cell wall. *Fg*Ste2 and *Fg*Ste3 are both predicted to contain sites for glycosylation in their extracellular N termini regions. Thus, the possibility that the receptors themselves display the peroxidase substrates cannot be ignored, however, more experiments are required to determine whether this is the case. Following their modification and release, these two products of the peroxidase-mediated reaction either independently bind to *Fg*Ste2 and *Fg*Ste3 to induce chemotropism or they become conjugated to form one ligand molecule. Peroxidases are well known to catalyze the polymerization (aka conjugation) of cell-wall-related metabolites (Hiraga, 2001; Passardi et al., 2004; Almagro et al., 2009; Cosio and Dunand, 2009). The precise identity of these substrates and the product generated by their peroxidase-mediated catalysis remain to be determined. Identifying these will provide essential information needed to understand the molecular mechanism underling the early stages of wheat infection by *F. graminearum* and how we might devise a way to control it.

## Data availability statement

The datasets presented in this study can be found in online repositories. The names of the repository/repositories and accession number(s) can be found in the article/**Supplementary Material**.

## Author contributions

PS: Formal analysis, Investigation, Methodology, Validation, Visualization, Writing – original draft. VV: Formal analysis, Investigation, Methodology, Writing – review & editing. FM: Formal analysis, Investigation, Methodology, Writing – review & editing. JA: Formal analysis, Funding acquisition, Methodology, Supervision, Writing – review & editing. ML: Conceptualization, Data curation, Formal analysis, Funding acquisition, Methodology, Supervision, Writing – review & editing.

## References

[B1] Almagro L. Gómez Ros L. V. Belchi-Navarro S. Bru R. Ros Barceló A. Pedreño M. A. (2009). Class III peroxidases in plant defence reactions. J. Exp. Bot. 60, 377–390. doi: 10.1093/jxb/ern277 19073963

[B2] Alvaro C. G. Thorner J. (2016). Heterotrimeric G protein-coupled receptor signaling in yeast mating pheromone response. J. Biol. Chem. 291, 7785–7798. doi: 10.1074/jbc.R116.714980 PMC4824985 26907689

[B3] Barbosa I. P. Kemmelmeier C. (1993). Chemical composition of the hyphal wall from fusarium graminearum. Exp. Mycology 17, 274–283. doi: 10.1006/emyc.1993.1026

[B4] Barkai N. Rose M. D. Wingreen N. S. (1998). Protease helps yeast find mating partners. Nature 396, 422–423. doi: 10.1038/24760 9853747

[B5] Basenko E. Y. Pulman J. A. Shanmugasundram A. Harb O. S. Crouch K. Starns D.. (2018). FungiDB: an integrated bioinformatic resource for fungi and oomycetes. J. Fungi (Basel) 4. doi: 10.3390/jof4010039 PMC5872342 30152809

[B6] Becker D. M. Guarente L. B. T.-M. (1991). High-efficiency transformation of yeast by electroporation, in: Guide to Yeast Genetics and Molecular Biology (Academic Press), 182–187. doi: 10.1016/0076-6879(91)94015-5 2005786

[B7] Benjamini Y. Hochberg Y. (1995). Controlling the false discovery rate: A practical and powerful approach to multiple testing. J. R. Stat. Society: Ser. B (Methodological) 57, 289–300. doi: 10.1111/j.2517-6161.1995.tb02031.x

[B8] Blumer K. J. Reneke J. E. Thorner J. (1988). The STE2 gene product is the ligand-binding component of the alpha-factor receptor of Saccharomyces cerevisiae. J. Biol. Chem. 263, 10836–10842. 2839507

[B9] Boylan M. T. Mirabito P. M. Willett C. E. Zimmerman C. R. Timberlake W. E. (1987). Isolation and physical characterization of three essential conidiation genes from Aspergillus nidulans. Mol. Cell. Biol. 7, 3113–3118. doi: 10.1128/mcb.7.9.3113-3118.1987 2823119 PMC367944

[B10] Bradford M. M. (1976). A rapid and sensitive method for the quantitation of microgram quantities of protein utilizing the principle of protein-dye binding. Analytical Biochem. 72, 248–254. doi: 10.1016/0003-2697(76)90527-3 942051

[B11] Brizzio V. Gammie A. E. Nijbroek G. Michaelis S. Rose M. D. (1996). Cell fusion during yeast mating requires high levels of a-factor mating pheromone. J. Cell Biol. 135, 1727–1739. doi: 10.1083/jcb.135.6.1727 8991086 PMC2133945

[B12] Cambaza E. (2018). Comprehensive description of fusarium graminearum pigments and related compounds. Foods 7, 165. doi: 10.3390/foods7100165 30301164 PMC6209861

[B13] Chang H.-X. Yendrek C. R. Caetano-Anolles G. Hartman G. L. (2016). Genomic characterization of plant cell wall degrading enzymes and in silico analysis of xylanases and polygalacturonases of Fusarium virguliforme. BMC Microbiol. 16, 147. doi: 10.1186/s12866-016-0761-0 27405320 PMC4941037

[B14] Chou C.-S. Bardwell L. Nie Q. Yi T.-M. (2011). Noise filtering tradeoffs in spatial gradient sensing and cell polarization response. BMC Syst. Biol. 5, 196. doi: 10.1186/1752-0509-5-196 22166067 PMC3268761

[B15] Choudhary P. Loewen M. C. (2016a). Quantification of mutation-derived bias for alternate mating functionalities of the Saccharomyces cerevisiae Ste2p pheromone receptor. J. Biochem. 159, 49–58. doi: 10.1093/jb/mvv072 26232403 PMC4882639

[B16] Choudhary P. Loewen M. C. (2016b). Evidence of a role for S. cerevisiae α-arrestin Art1 (Ldb19) in mating projection and zygote formations: A role for yeast arrestin-1(Ldb19) in mating. Cell Biol. Int. 40, 83–90. doi: 10.1002/cbin.10541 26314564

[B17] Cosio C. Dunand C. (2009). Specific functions of individual class III peroxidase genes. J Exp Bot. 60 (2), 391–408. doi: 10.1093/jxb/ern318 19088338

[B18] Deshpande N. Wilkins M. R. Packer N. Nevalainen H. (2008). Protein glycosylation pathways in filamentous fungi. Glycobiology 18, 626–637. doi: 10.1093/glycob/cwn044 18504293

[B19] Dube P. Konopka J. B. (1998). Identification of a polar region in transmembrane domain 6 that regulates the function of the G protein-coupled alpha-factor receptor. Mol. Cell. Biol. 18, 7205–7215. doi: 10.1128/MCB.18.12.7205 9819407 PMC109302

[B20] Elia L. (1996). Role of the ABC transporter Ste6 in cell fusion during yeast conjugation. J. Cell Biol. 135, 741–751. doi: 10.1083/jcb.135.3.741 8909547 PMC2121058

[B21] Foata F. Sprenger N. Rochat F. Damak S. (2020). Activation of the G-protein coupled receptor GPR35 by human milk oligosaccharides through different pathways. Sci. Rep. 10, 16117. doi: 10.1038/s41598-020-73008-0 32999316 PMC7528069

[B22] Futagami T. Nakao S. Kido Y. Oka T. Kajiwara Y. Takashita H.. (2011). Putative Stress Sensors WscA and WscB Are Involved in Hypo-Osmotic and Acidic pH Stress Tolerance in Aspergillus nidulans. Eukaryotic Cell 10, 1504–1515. doi: 10.1128/EC.05080-11 21926329 PMC3209062

[B23] Ghanegolmohammadi F. Okada H. Liu Y. Itto-Nakama K. Ohnuki S. Savchenko A.. (2021). Defining functions of mannoproteins in saccharomyces cerevisiae by high-dimensional morphological phenotyping. JoF 7, 769. doi: 10.3390/jof7090769 34575807 PMC8466635

[B24] Glass N. L. Kaneko I. (2003). Fatal attraction: nonself recognition and heterokaryon incompatibility in filamentous fungi. Eukaryotic Cell 2, 1–8. doi: 10.1128/EC.2.1.1-8.2003 12582117 PMC141178

[B25] Hagen D. C. McCaffrey G. Sprague G. F. J. (1986). Evidence the yeast STE3 gene encodes a receptor for the peptide pheromone a factor: gene sequence and implications for the structure of the presumed receptor. Proc. Natl. Acad. Sci. U.S.A. 83, 1418–1422. doi: 10.1073/pnas.83.5.1418 3006051 PMC323087

[B26] Hartwell L. H. (1980). Mutants of Saccharomyces cerevisiae unresponsive to cell division control by polypeptide mating hormone. J. Cell Biol. 85, 811–822. doi: 10.1083/jcb.85.3.811 6993497 PMC2111434

[B27] Hilger D. Masureel M. Kobilka B. K. (2018). Structure and dynamics of GPCR signaling complexes. Nat. Struct. Mol. Biol. 25, 4–12. doi: 10.1038/s41594-017-0011-7 29323277 PMC6535338

[B28] Hinterdobler W. Li G. Turrà D. Schalamun M. Kindel S. Sauer U.. (2021). Integration of chemosensing and carbon catabolite repression impacts fungal enzyme regulation and plant associations. BioRxiv. doi: 10.1101/2021.05.06.442915

[B29] Hiraga S. (2001). A large family of class III plant peroxidases. Plant Cell Physiol. 42, 462–468. doi: 10.1093/pcp/pce061 11382811

[B30] Hsueh Y.-P. Xue C. Heitman J. (2009). A constitutively active GPCR governs morphogenic transitions in Cryptococcus neoformans. EMBO J. 28, 1220–1233. doi: 10.1038/emboj.2009.68 19322200 PMC2683048

[B31] Jackson C. L. Konopka J. B. Hartwell L. H. (1991). S. cerevisiae α pheromone receptors activate a novel signal transduction pathway for mating partner discrimination. Cell 67, 389–402. doi: 10.1016/0092-8674(91)90190-A 1655282

[B32] Jones S. K. Bennett R. J. (2011). Fungal mating pheromones: Choreographing the dating game. Fungal Genet. Biol. 48, 668–676. doi: 10.1016/j.fgb.2011.04.001 21496492 PMC3100450

[B33] King R. Urban M. Hammond-Kosack M. C. U. Hassani-Pak K. Hammond-Kosack K. E. (2015). The completed genome sequence of the pathogenic ascomycete fungus Fusarium graminearum. BMC Genomics 16, 544. doi: 10.1186/s12864-015-1756-1 26198851 PMC4511438

[B34] King R. Urban M. Lauder R. P. Hawkins N. Evans M. Plummer A.. (2017). A conserved fungal glycosyltransferase facilitates pathogenesis of plants by enabling hyphal growth on solid surfaces. PLoS Pathog. 13, 1–26. doi: 10.1371/journal.ppat.1006672 PMC5653360 29020037

[B35] Krumm B. E. Lee S. Bhattacharya S. Botos I. White C. F. Du H.. (2016). Structure and dynamics of a constitutively active neurotensin receptor. Sci. Rep. 6, 38564. doi: 10.1038/srep38564 27924846 PMC5141500

[B36] Lamichhane R. Liu J. J. Pljevaljcic G. White K. L. van der Schans E. Katritch V.. (2015). Single-molecule view of basal activity and activation mechanisms of the G protein-coupled receptor β2AR. Proc. Natl. Acad. Sci. 112, 14254–14259. doi: 10.1073/pnas.1519626112 26578769 PMC4655547

[B37] Latorraca N. R. Venkatakrishnan A. J. Dror R. O. (2017). GPCR dynamics: structures in motion. Chem. Rev. 117, 139–155. doi: 10.1021/acs.chemrev.6b00177 27622975

[B38] Lee H. Damsz B. Woloshuk C. P. Bressan R. A. Narasimhan M. L. (2010). Use of the plant defense protein osmotin to identify fusarium oxysporum genes that control cell wall properties. Eukaryot Cell 9, 558–568. doi: 10.1128/EC.00316-09 20190074 PMC2863404

[B39] Lemaire K. Van de Velde S. Van Dijck P. Thevelein J. M. (2004). Glucose and sucrose act as agonist and mannose as antagonist ligands of the G protein-coupled receptor gpr1 in the yeast saccharomyces cerevisiae. Mol. Cell 16, 293–299. doi: 10.1016/j.molcel.2004.10.004 15494315

[B40] Li X. Staszewski L. Xu H. Durick K. Zoller M. Adler E. (2002). Human receptors for sweet and umami taste. Proc. Natl. Acad. Sci. U.S.A. 99, 4692–4696. doi: 10.1073/pnas.072090199 11917125 PMC123709

[B41] Lodder A. L. Lee T. K. Ballester R. (1999). Characterization of the Wsc1 protein, a putative receptor in the stress response of Saccharomyces cerevisiae. Genetics 152, 1487–1499. doi: 10.1093/genetics/152.4.1487 10430578 PMC1460702

[B42] Lu S. Jang W. Inoue A. Lambert N. A. (2021). Constitutive G protein coupling profiles of understudied orphan GPCRs. PLoS One 16, e0247743. doi: 10.1371/journal.pone.0247743 33886554 PMC8062009

[B43] Maehly A. C. Chance B. (1954). The assay of catalases and peroxidases. Methods Biochem Anal. 1, 357–424. doi: 10.1002/9780470110171.ch14 13193536

[B44] Martín-Urdíroz M. Roncero M. I. G. González-Reyes J. A. Ruiz-Roldán C. (2008). ChsVb, a class VII chitin synthase involved in septation, is critical for pathogenicity in *fusarium oxysporum* . Eukaryot Cell 7, 112–121. doi: 10.1128/EC.00347-07 17993572 PMC2224148

[B45] Meye F. J. Ramakers G. M. J. Adan R. A. H. (2014). The vital role of constitutive GPCR activity in the mesolimbic dopamine system. Trans. Psychiatry 4, e361–e361. doi: 10.1038/tp.2013.130 PMC3944632 24518399

[B46] Mirabito P. M. Adams T. H. Timberlake W. E. (1989). Interactions of three sequentially expressed genes control temporal and spatial specificity in aspergillus development. Cell 57, 859–868. doi: 10.1016/0092-8674(89)90800-3 2655931

[B47] Nakashima A. Takeuchi H. Imai T. Saito H. Kiyonari H. Abe T.. (2013). Agonist-independent GPCR activity regulates anterior-posterior targeting of olfactory sensory neurons. Cell 154, 1314–1325. doi: 10.1016/j.cell.2013.08.033 24034253 PMC7394037

[B48] Nordzieke D. E. Fernandes T. R. El Ghalid M. Turrà D. Di Pietro A. (2019). NADPH oxidase regulates chemotropic growth of the fungal pathogen *Fusarium oxysporum* towards the host plant. New Phytol. 224, 1600–1612. doi: 10.1111/nph.16085 31364172

[B49] Ohsawa S. Yurimoto H. Sakai Y. (2017). Novel function of Wsc proteins as a methanol-sensing machinery in the yeast Pichia pastoris. Mol. Microbiol. 104, 349–363. doi: 10.1111/mmi.13631 28127815

[B50] Pandey V. P. Awasthi M. Singh S. Tiwari S. Dwivedi U. N. (2017). A comprehensive review on function and application of plant peroxidases. Biochem. Anal. Biochem. 6, 1–16. doi: 10.4172/2161-1009.1000308

[B51] Passardi F. Penel C. Dunand C. (2004). Performing the paradoxical: How plant peroxidases modify the cell wall. Trends Plant Sci. 9, 534–540. doi: 10.1016/j.tplants.2004.09.002 15501178

[B52] Rampitsch C. Leung W. W. Y. Blackwell B. A. Subramaniam R. (2011). MAP kinase Mgv1: a potential shared control point of butenolide and deoxynivalenol biosynthesis in Fusarium graminearum. Plant Breed. Seed Sci. 54, 81–88. doi: 10.2478/v10129-011-0031-0

[B53] Saupe S. J. (2000). Molecular genetics of heterokaryon incompatibility in filamentous ascomycetes. Microbiol. Mol. Biol. reviews : MMBR 64, 489–502. doi: 10.1128/MMBR.64.3.489-502.2000 10974123 PMC99001

[B54] Schrick K. Garvik B. Hartwell L. H. (1997). Mating in Saccharomyces cerevisiae: The role of the pheromone signal transduction pathway in the chemotropic response to pheromone. Genetics 147, 19–32. doi: 10.1093/genetics/147.1.19 9286665 PMC1208103

[B55] Schweikert C. Liszkay A. Schopfer P. (2002). Polysaccharide degradation by Fenton reaction- or peroxidase-generated hydroxyl radicals in isolated plant cell walls. Phytochemistry 61, 31–35. doi: 10.1016/S0031-9422(02)00183-8 12165299

[B56] Segall J. E. (1993). Polarization of yeast cells in spatial gradients of alpha mating factor. Proc. Natl. Acad. Sci. U.S.A. 90, 8332–8336. doi: 10.1073/pnas.90.18.8332 8397402 PMC47350

[B57] Seong K. Y. Zhao X. Xu J. R. Güldener U. Kistler H. C. (2008). Conidial germination in the filamentous fungus Fusarium graminearum. Fungal Genet. Biol. 45, 389–399. doi: 10.1016/j.fgb.2007.09.002 17950638

[B58] Sewall T. C. (1994). Cellular effects of misscheduled brlA, abaA and wetA expression in Aspergillus nidulans. Can. J. Microbiol. 40, 1035–1042. doi: 10.1139/m94-164 7704830

[B59] Sewall T. C. Mims C. W. Timberlake W. E. (1990). abaA controls phialide differentiation in aspergillus nidulans. Plant Cell 2, 731–739. doi: 10.2307/3869172 2152124 PMC159926

[B60] Sharma T. Sridhar P. S. Blackman C. Foote S. J. Allingham J. S. Subramaniam R.. (2022). *Fusarium graminearum* ste3 G-protein coupled receptor: A mediator of hyphal chemotropism and pathogenesis. mSphere 7, e0045622. doi: 10.1128/msphere.00456-22 36377914 PMC9769807

[B61] Shi C. Kaminskyj S. Caldwell S. Loewen M. C. (2007). A role for a complex between activated G protein-coupled receptors in yeast cellular mating. Proc. Natl. Acad. Sci. 104, 5395–5400. doi: 10.1073/pnas.0608219104 17369365 PMC1838501

[B62] Shi C. Kendall S. C. Grote E. Kaminskyj S. Loewen M. C. (2009). N-terminal residues of the yeast pheromone receptor, Ste2p, mediate mating events independently of G1-arrest signaling. J. Cell. Biochem. 107, 630–638. doi: 10.1002/jcb.22129 19459151

[B63] Son H. Kim M.-G. Min K. Lim J. Y. Choi G. J. Kim J.-C.. (2014). WetA is required for conidiogenesis and conidium maturation in the ascomycete fungus fusarium graminearum. Eukaryot Cell 13, 87–98. doi: 10.1128/EC.00220-13 24186953 PMC3910957

[B64] Son H. Kim M.-G. Min K. Seo Y.-S. Lim J. Y. Choi G. J.. (2013). AbaA regulates conidiogenesis in the ascomycete fungus fusarium graminearum. PLoS One 8, e72915. doi: 10.1371/journal.pone.0072915 24039821 PMC3769392

[B65] Sridhar P. S. Trofimova D. Subramaniam R. González-Peña Fundora D. Foroud N. A. Allingham J. S.. (2020). Ste2 receptor-mediated chemotropism of Fusarium graminearum contributes to its pathogenicity against wheat. Sci. Rep. 10, 10770. doi: 10.1038/s41598-020-67597-z 32612109 PMC7329813

[B66] Stajich J. E. Harris T. Brunk B. P. Brestelli J. Fischer S. Harb O. S.. (2012). FungiDB: an integrated functional genomics database for fungi. Nucleic Acids Res. 40, D675–D681. doi: 10.1093/nar/gkr918 22064857 PMC3245123

[B67] Turrà D. El Ghalid M. Rossi F. Di Pietro A. (2015). Fungal pathogen uses sex pheromone receptor for chemotropic sensing of host plant signals. Nature 527, 521–524. doi: 10.1038/nature15516 26503056

[B68] Vangalis V. Markakis E. A. Knop M. Di Pietro A. Typas M. A. Papaioannou I. A. (2022). Components of TOR and MAP kinase signaling control chemotropism and pathogenicity in the fungal pathogen Verticillium dahliae. bioRxiv 06, 20. doi: 10.1101/2022.06.20.496898 36921400

[B69] Varet H. Brillet-Guéguen L. Coppée J.-Y. Dillies M.-A. (2016). SARTools: A DESeq2- and edgeR-based R pipeline for comprehensive differential analysis of RNA-seq data. PLoS One 11, e0157022. doi: 10.1371/journal.pone.0157022 27280887 PMC4900645

[B70] Verna J. Lodder A. Lee K. Vagts A. Ballester R. (1997). A family of genes required for maintenance of cell wall integrity and for the stress response in Saccharomyces cerevisiae. Proc. Natl. Acad. Sci. United States America 94, 13804–13809. doi: 10.1073/pnas.94.25.13804 PMC28388 9391108

[B71] Vitale S. Di Pietro A. Turrà D. (2019). Autocrine pheromone signalling regulates community behaviour in the fungal pathogen Fusarium oxysporum. Nat. Microbiol. 4, 1443–1449. doi: 10.1038/s41564-019-0456-z 31133754

[B72] Wang T. Cai Z. P. Gu X. Q. Ma H. Y. Du Y. M. Huang K.. (2014). Discovery and characterization of a novel extremely acidic bacterial *N* -glycanase with combined advantages of PNGase F and A. Bioscience Rep. 34, e00149. doi: 10.1042/BSR20140148 PMC4231336 25294009

[B73] Weis W. I. Kobilka B. K. (2018). The molecular basis of G protein–coupled receptor activation. Annu. Rev. Biochem. 87, 897–919. doi: 10.1146/annurev-biochem-060614-033910 29925258 PMC6535337

[B74] Wilde C. Fischer L. Lede V. Kirchberger J. Rothemund S. Schöneberg T.. (2016). The constitutive activity of the adhesion GPCR GPR114/ADGRG5 is mediated by its tethered agonist. FASEB J. 30, 666–673. doi: 10.1096/fj.15-276220 26499266

[B75] Winzeler E. A. Shoemaker D. D. Astromoff A. Liang H. Anderson K. Andre B.. (1999). Functional characterization of the S. cerevisiae genome by gene deletion and parallel analysis. Science 285, 901–906. doi: 10.1126/science.285.5429.901 10436161

[B76] Yang B. Y. Gray J. S. S. Montgomery R. (1996). The glycans of horseradish peroxidase. Carbohydr. Res. 287, 203–212. doi: 10.1016/0008-6215(96)00073-0 8766207

[B77] Yun Y. Liu Z. Zhang J. Shim W. B. Chen Y. Ma Z. (2014). The MAPKK FgMkk1 of Fusarium graminearum regulates vegetative differentiation, multiple stress response, and virulence via the cell wall integrity and high-osmolarity glycerol signaling pathways. Environ. Microbiol. 16, 2023–2037. doi: 10.1111/1462-2920.12334 24237706

