# Supplementary material for: A peroxidase-derived ligand that induces Fusarium graminearum Ste2 receptor-dependent chemotropism
Source: Front Cell Infect Microbiol. 2024 Jan 4;13:1287418. doi: 10.3389/fcimb.2023.1287418 (PMC10794396; doi:10.3389/fcimb.2023.1287418)
Supplement: Supplementary file 2 [file DataSheet_1.pdf]

Supplementary Material

A Peroxidase-derived Ligand that Induces *Fusarium graminearum* Ste2 Receptor-dependent Chemotropism

Pooja S. Sridhar[1], Vinicio Vasquez[2], Fanny Monteil-Rivera[2], John S. Allingham[1], Michele C. Loewen[1,3]

[1]Department of Biomedical and Molecular Sciences, Queen's University, Kingston, Canada, K7L 3N6

[2]National Research Council of Canada, Aquatic and Crop Resources Development, Montreal, Quebec, Canada, H4P 2R2

[3]National Research Council of Canada, Aquatic and Crop Resources Development, Ottawa, Ontario, Canada, K1A 0R6

Supplementary Figure S1

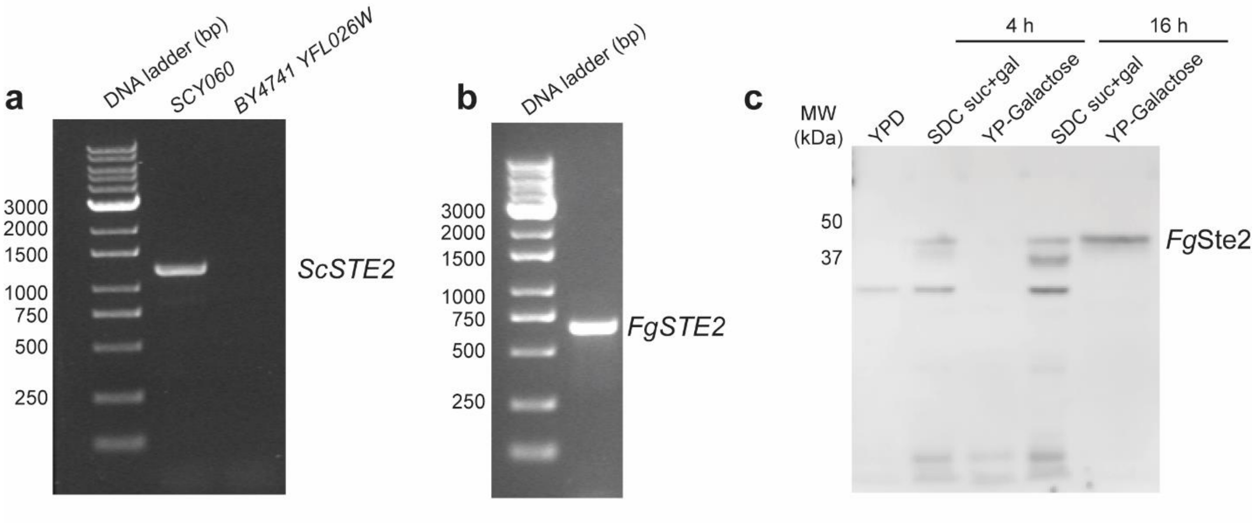

Figure S1. Peroxidase treatment of *Fg*Ste2-expressing *S. cerevisiae* cells results in extraction of *Fg*Ste2-activating ligand. a. Polymerase chain reaction using primers internal to the *ScSTE2* gene was used to verify the absence of the native *ScSTE2* gene in the *BY4741 YFL026W* strain. A different wild-type strain (*SCY060*) was used as a positive control for the reaction. Expected size of the amplified fragment is 1241 bp. **b.** Polymerase chain reaction was used to confirm the successful transformation of pYESDEST52-*FgSTE2* into the *BY4741 YFL026W* strain using primers internal to the *FgSTE2* gene. Expected size of the amplified fragment is 657 bp. **c.** Representative Western blot showing expression of His-tagged *Fg*Ste2 in *S. cerevisiae*. Samples were probed for using an anti-6x His antibody. The expected molecular weight of His-tagged *Fg*Ste2 is 42 kDa.
